# Cinnamomeoventrolide – Double Bond Regioisomerism in Frog Semiochemicals

**DOI:** 10.1007/s10886-022-01370-6

**Published:** 2022-07-09

**Authors:** Johanna Kuhn, Stefan Schulz

**Affiliations:** Institute of Organic Chemistry, Technische Universität Braunschweig, Hagenring 30, Braunschweig, 38106 Germany

**Keywords:** Anurans, Enantioselective synthesis, Frog semiochemicals, Macrocyclic lactones, Mass spectra, Pheromones

## Abstract

Frogs of the families Mantellinae and Hyperoliidae possess male specific femoral or gular glands that are used during courtship. These glands release volatile compounds, e. g. the macrocyclic lactone gephyromantolide A (2,6,10-trimethyl-6-undecen-11-olide) in the case of *Gephyromantis boulengeri* (Mantellinae). During the analysis of the volatiles of *Hyperolius cinnamomeoventris* (Hyperoliidae) we detected an unknown compound **A**, which we called cinnamomeoventrolide, whose mass spectrum showed high similarity with the spectrum of gephyromantolide A. Nevertheless, slight spectral differences led to the proposal of a regioisomer of gephyromantolide A as a structure for **A**, 2,6,10-trimethyl-5-undecen-11-olide. A versatile synthesis of this compound was developed to allow access to all four stereoisomers from a single chiral starting material, the so-called (*S*)-Roche ester, using ring-closing metathesis as a key step. With these stereoisomers, the absolute configuration of the natural product was established to have the (2*R*,10*S*)-configuration by GC on a chiral phase. The configuration of natural gephyromantolide A is the opposite. Both frogs seem to use a similar biosynthetic pathway to access the target compounds, differing in the stereochemistry of the reduction steps, and requiring an additional isomerization in case of *G. boulengeri*. This unique regioisomeric differentiation of double bonds in semiochemicals has so far only been observed in insects. The compounds are likely to play a role in species-recognition of the frogs.

**Supplementary Information:**

The online version contains supplementary material available at 10.1007/s10886-022-01370-6.

## Introduction

**Fig. 1:**
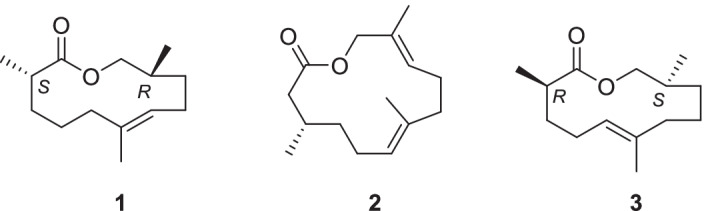
Structure of gephyromantolide A (**1**), frogolide (**2**), and cinnamomeoventrolide (**3**)

**Fig. 2:**
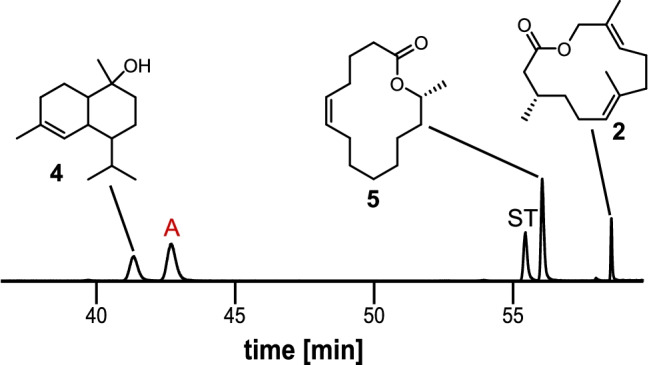
Total ion current chromatogram of a gular gland extract of *Hyperolius cinnamomeoventris* on a Hydrodex β-6TBDM phase. ST = unknown sesquiterpene, **A** = macrocyclic lactone

In recent years it became evident that frogs communicate using not only vocal cues or vision, but also by using volatile semiochemicals (Poth et al. 2012; Starnberger et al. 2013; Woodley 2015; Brunetti et al. 2016, 2019; Schulte 2016; Nowack et al. 2017; Glos et al. 2019; Graham et al. 2020; Deng et al. 2021). Some frog lineages, such as Mantellinae (Glaw et al. 2006) or Hyperoliidae (Starnberger et al. 2014b) have evolved male specific scent releasing structures, such as femoral or gular glands to disseminate volatile compounds. Although numerous mantelline and hyperolid species have been analyzed, the function of the volatile compounds is not well understood. In the mantelline *Mantidactylus multiplicatus*, the macrolide phoracantholide J and (*R*)-8-methyl-2-nonanol induces attraction of females and generally increases the activity of both sexes (Poth et al. 2012). The putative pheromone of *M. betsileanus*, phoracantholide J, can stimulate distinct sensory neurons in the main olfactory organ, while the vomeronasal organ is decoupled (Nowack et al. 2017). This decoupling is a derived state and occurs together with femoral gland occurrence in mantellid frogs (Nowack and Vences 2016). The volatiles released in the femoral and gular glands often comprise of species specific mixtures of various components including aliphatic macrocyclic lactones, terpenes, and various other aliphatic compounds such as alcohols, ketones or esters (Poth et al. 2013; Starnberger et al. 2013; Nowack et al. 2017; Schulz et al. 2021). The Madagascan frog *Gephyromantis boulengeri* is a somewhat unusual exception because its femoral glands contain almost exclusively gephyromantolide A (**1**) (Fig. 1), whose structure was elucidated by NMR spectroscopy of isolated material and by total synthesis (Poth et al. 2012). During the analysis of the gular gland constituents of the hyperolid frog *Hyperolius cinnamomeoventris*, not related to *G. boulengeri*, we detected a compound (**A** in Fig. 2) that showed a very similar mass spectrum and a similar gas chromatographic retention index as **1**, thus being tentatively identified as gephyromantolide A (Menke et al. 2016). The hydrogenation of both gephyromantolide A and compound **A** led to identical products, thus further corroborating this assignment.

Although gephyromantolide A has only 14 carbons, it likely originates from the terpene biosynthetic pathway (Schulz et al. 2021), losing one carbon by oxidation during its biosynthesis. The occurrence of **A** together with frogolide (**2**) (Fig. 1) in the gular gland of *H. cinnamomeoventris* (Fig. 2) further supported that **A **is biosynthesized via the terpene pathway (Menke et al. 2018). Nevertheless, a close inspection of the mass spectra of **1** and **A** showed some minor differences. Therefore, we assumed **A** to be a regioisomer of **1**, with the double bond at the generic terpene position at C-5, in contrast to the C-6-double bond found in **1**. In the following sections we describe the structural elucidation, synthesis, and determination of the absolute configuration of **A**, which we have called cinnamomeoventrolide (**3**) (Fig. 1).

## Methods and Materials

### Biological Material

The samples obtained from gular gland extracts of *H. cinnamomeoventris* previously described by Starnberger et al. (2013) were used in this study.

### General Experimental Procedures

Chemicals were obtained from commercial suppliers and used without further purification unless otherwise noted. All reactions were performed in oven-dried glassware under a nitrogen atmosphere. Solvents were dried according to standard procedures. Column chromatography was performed on silica gel 60 (Fluka, particle size 0.040–0.063 mm, mesh 230–440 ASTM) and thin-layer chromatography (TLC) on Polygram® SIL G/UV_254_ silica 60 plates (Macherey & Nagel, 0.20 mm thickness). Compounds were detected with UV light (254 nm), potassium permanganate or phosphomolybdic acid staining solutions, followed by heating. Argentation column chromatography was performed with silica gel impregnated with 10% silver nitrate according to a procedure by Li et al*.* (1995). Derivatizations with *N*-methyl-*N*-trimethylsilyltrifluoroacetamide (MSTFA) were performed by adding 50 μl MSTFA to the sample (5 mg) in dichloromethane (DCM, 1 ml) and heating the mixture at 60 °C for one h. The excess solvent and other volatile components were removed in a gentle stream of nitrogen and the residue was taken up in DCM. NMR analyses were performed on Bruker Avance III HD 300 N (300 MHz for ^1^H, 75 MHz for ^13^C) and Avance III HD 500 (500 MHz for ^1^H, 125 MHz for ^13^C) spectrometers at room temperature. Chemical shifts are reported in ppm from tetramethylsilane as an internal standard (δ = 0). Multiplicities of the protons are described as singlets (s), doublets (d), triplets (t), quartets (q), quintets (quint), sextets (sext), septets (sept), or multiplets (m). The multiplicities of the carbon atoms are described as primary (CH_3_), secondary (CH_2_), tertiary (CH), or quaternary (C_q_). GC/MS analyses of synthetic products were performed with a combination of an Agilent Technologies 5977B gas chromatograph connected to an Agilent Technologies 8860 Series MSD. Mass spectrometry was performed in electron ionization mode (EI) with 70 eV. A HP-5 MS column (Agilent Technologies, 30 m length, 0.25 mm diameter, 0.25 μm film thickness, 350 °C) with helium as carrier gas was used. The temperature program started at 50 °C, which was held for five mins, followed by an increase with a rate of 20 °C/min to 320 °C. GC analyses on a chiral phase were performed using the combination of an Agilent Technologies 7890A with an Agilent Technologies 5975 Series MSD. A Hydrodex β-6TBDM column (Macherey & Nagel, 25 m length, 0.25 mm diameter, 0.25 μm film thickness, 230 °C) with helium as carrier gas was used for the separation. The temperature program started at 50 °C, and was held for five mins. After that, the oven was heated to 110 °C at a rate of 20 °C/min and the temperature was maintained for 45 min. Finally, the temperature was increased to 230 °C at a rate of 10 °C/min for improvement of the peak shape and the final temperature was maintained for 25 min. IR spectra were recorded on a Bruker Tensor 27 (diamond ATR). The intensities of the bands are given as strong (s), medium (m), weak (w) or broad (br). Optical rotations were determined with an MCP 150 polarimeter (Anton Paar) with a cell length of 10 cm, 589 nm at 25 °C (c given in g/100 ml).

### Synthetic Procedures

#### General Procedure A for Reduction with NaBH_4_

Sodium borohydride (2.2 equiv) and boron trifluoride diethyl etherate (0.3 equiv) were added at 0 °C to a solution of the ester (1.0 equiv) in dry and degassed tetrahydrofuran (THF, 0.35 M) (Zhang and Li 2018). The reaction mixture was stirred at rt and monitored by TLC. After completion, the reaction was quenched with sat. aq. NaHCO_3_ solution and diethyl ether was added. The phases were separated, and the aq. layer was extracted with diethyl ether (3 times). The comb. org. layers were dried over anhydrous MgSO_4_, filtered, and concentrated under reduce pressure. The residue was purified by column chromatography.

#### General Procedure B for Oxidation using the Corey-Schmidt Method

Pyridinium dichromate (PDC) (5.0 equiv) was added to a solution of the alcohol (1.0 equiv) in *N*,*N*-dimethylformamide (DMF) (0.2 M) at rt (Peram et al. 2017). After stirring for 24 h at rt, the reaction mixture was filtered through Celite®, and the filter cake was washed with diethyl ether. The filtrate was washed with 1 M HCl, the phases were separated, and the aq. layer was extracted with diethyl ether (3 times). The comb. org. layers were washed with brine, dried over anhydrous MgSO_4_, filtered, and concentrated under reduce pressure. The residue was purified by column chromatography.

#### General Procedure C for Coupling with Gilman-cuprate

The iodide (9.5 equiv) was dissolved in a 3:2 mixture of dry hexane and diethyl ether (0.5 M, degassed using the freeze pump thaw method) (Nurdin et al. 2020). Then *t*-butyllithium (**Attention: careful handling required, extremely flammable reagent,** 19.0 equiv) was added at –78 °C and the reaction mixture was stirred at this temperature for 1.5 h. The obtained organolithium solution was used in the next step without further purification. A solution of copper cyanide (4.75 equiv) in dry diethyl ether (0.3 M) was slowly added the organolithium solution at –78 °C (Chiou and Chen 2017). The reaction mixture was allowed to warm to –10 °C over 2 h and then recooled to –78 °C. Then a solution of tosylate (1.0 equiv) in dry diethyl ether (0.14 M) was slowly added and the reaction mixture was allowed to warm to rt over 3 h and stirred for 16 h at rt. The reaction mixture was quenched with aq. ammonia solution, water, and diethyl ether were added and the phases were separated. The aq. layer was extracted with diethyl ether (3 times), the comb. org. layers were dried over anhydrous MgSO_4_, filtered, and concentrated under reduced pressure. The residue was purified by column chromatography.

#### General Procedure D for Deprotection of Silyl ethers

Tetra-*n*-butylammonium fluoride (TBAF, 1.2 equiv) was added to a solution of the silyl ether (1.0 equiv) in dry THF (0.3 M) at 0 °C (Dash et al. 2015). After stirring for 18 h at rt, the reaction mixture was quenched with sat. aq. NaHCO_3_ solution, diethyl ether was added, and the phases were separated. The aq. layer was extracted with diethyl ether (3 times), the comb. org. layers were washed with brine, dried over anhydrous MgSO_4_, filtered, and concentrated under reduce pressure. The residue was purified by column chromatography.

#### General Procedure E for Esterification

The alcohol (1.0 equiv) and the carboxylic acid (1.0 equiv) were dissolved in dry DCM (0.2 M), followed by addition of 4-(dimethylamino)pyridine (DMAP, 10 mol%) and 1-ethyl-3-(3-dimethylaminopropyl)carbodiimide-hydrochloride (EDC-HCl, 1.1 equiv) in portions at 0 °C (Chinta et al. 2016). After stirring for one h at 0 °C and 18 h at rt, the reaction mixture was quenched with water, and DCM was added. The phases were separated, and the aq. layer was extracted with DCM (3 times). The comb. org. layers were washed with brine, dried over anhydrous MgSO_4_, filtered, and concentrated under reduce pressure. The residue was purified by column chromatography (silica gel, pentane/diethyl ether 20:1) to afford the ester.

#### General Procedure F for Ring-Closing Metathesis

Hexafluorobenzene (10 equiv), tetrafluoro-*p*-benzoquinone (10 mol%), and benzylidene[1,3-bis(2,4,6-trimethylphenyl)-2-imidazolidinylidene]dichloro(tricyclohexylphosphine)ruthenium (Grubbs II generation catalyst, 10 mol%) were added to a solution of the ester (1.0 equiv) in dry toluene (0.8 mM, degassed using the freeze pump thaw method) (Peram et al. 2017). After stirring for 6 h at 80 °C, further Grubbs catalyst (10 mol%) was added and the reaction mixture was stirred for 16 h at 80 °C. The catalyst was removed by filtration over a short column (8 cm silica gel filling height, diethyl ether) and the filtrate was washed with sat. aq. NaHCO_3_ solution. The phases were separated, and the aq. layer was extracted with diethyl ether (3 times). The comb. org. layers were dried over anhydrous MgSO_4_, filtered, and concentrated under reduced pressure. The residue was purified by column chromatography (silica gel, pentane/diethyl ether 40:1) to afford a mixture of the *E*/*Z* isomers, followed by silver ion chromatography leading to products highly enriched in the *E*-isomer.

**Preparation of methyl** **(***S***)-2-methyl-3-(tosyloxy)propanoate (7).** Triethylamine (2.45 ml, 1.79 g, 17.7 mmol, 1.3 equiv), DMAP (249 mg, 2.04 mmol, 0.15 equiv) and tosyl chloride (3.11 g, 16.3 mmol, 1.2 equiv) were added sequentially to a solution of methyl (*S*)-3-hydroxy-2-methylpropanoate ((*S*)-Roche ester, **6**, 1.61 g, 13.6 mmol, 1.0 equiv) in dry DCM (20 ml) at 0 °C (Han 2018). After stirring for 4.5 h at rt, water (100 ml) was added, the phases were separated, and the aq. layer was extracted with diethyl ether (3 × 50 ml). The comb. org. layers were washed with brine (100 ml), dried over anhydrous Na_2_SO_4_, filtered and concentrated under reduce pressure. The residue was purified by column chromatography (silica gel, pentane/diethyl ether 1:1) to afford methyl (*S*)-2-methyl-3-(tosyloxy)propanoate (**7**) as a colorless oil (2.94 g, 10.8 mmol, 79%). R_*f*_ = 0.39 (pentane/diethyl ether 1:1); ${[\mathrm{\alpha }]}_{\mathrm{D}}^{25}$ =  + 11.9 (c = 1.00, DCM); ^1^H NMR (300 MHz, CDCl_3_) δ 7.81–7.76 (m, 2H, CH_Ar_), 7.38–7.33 (m, 2H, CH_Ar_), 4.19 (dd, *J* = 9.7, 6.8 Hz, 1H, C*H*_a_H_b_), 4.06 (dd, *J* = 9.7, 6.1 Hz, 1H, CH_a_*H*_b_), 3.65 (s, 3H, CH_3_), 2.88–2.75 (m, 1H, CH), 2.46 (s, 3H, CH_3_), 1.17 (d, *J* = 7.2 Hz, 3H, CH_3_); ^13^C NMR (75 MHz, CDCl_3_) δ 173.1 (C_q_), 144.9 (C_q_), 132.7 (C_q_), 129.8 (2C, CH), 128.0 (2C, CH), 70.7 (CH_2_), 52.0 (CH_3_), 39.2 (CH), 21.6 (CH_3_), 13.6 (CH_3_); EIMS *m*/*z* (%) 272 (3) [M]^+^, 187 (5), 172 (6), 155 (58), 139 (3), 117 (42), 107 (7), 101 (4), 91 (100), 85 (21), 77 (5), 65 (32), 59 (15), 51 (4), 41 (10).

**Preparation of (***R***)-3-hydroxy-2-methylpropyl 4-methylbenzenesulfonate (8).** Ester **7** (100 mg, 0.367 mmol, 1.0 equiv) was reduced to alcohol **8** using sodium borohydride (30.5 mg, 0.807 mmol, 2.2 equiv) and boron trifluoride diethyl etherate (0.01 ml, 15.6 mg, 0.110 mmol, 0.3 equiv) in dry and degassed THF (1 ml) by following the general procedure A. The reaction mixture was stirred for 18 h at rt. The crude product was purified by column chromatography (silica gel, pentane/diethyl ether 1:1 → 1:5) to afford (*R*)-3-hydroxy-2-methylpropyl 4-methylbenzenesulfonate (**8**) as a colorless liquid (80.0 mg, 0.327 mmol, 89%). R_*f*_ = 0.46 (pentane/diethyl ether 1:2); ${[\mathrm{\alpha }]}_{\mathrm{D}}^{25}$ = –4.8 (c = 1.00, DCM); ^1^H NMR (300 MHz, CDCl_3_) δ 7.83–7.76 (m, 2H, CH_Ar_), 7.39–7.32 (m, 2H, CH_Ar_), 4.07–3.97 (m, 2H, CH_2_), 3.59 (dd, *J* = 11.0, 5.1 Hz, 1H, C*H*_a_H_b_), 3.51 (dd, *J* = 11.0, 6.5 Hz, 1H, CH_a_*H*_b_), 2.45 (s, 3H, CH_3_), 2.08–1.93 (m, 1H, CH), 1.76 (br. s, 1H, OH), 0.92 (d, *J* = 7.0 Hz, 3H, CH_3_); ^13^C NMR (75 MHz, CDCl_3_) δ 144.8 (C_q_), 132.9 (C_q_), 129.9 (2C, CH), 127.9 (2C, CH), 71.9 (CH_2_), 63.6 (CH_2_), 35.5 (CH), 21.6 (CH_3_), 13.0 (CH_3_); EIMS *m*/*z* (%) 203 (8), 173 (75), 155 (28), 139 (2), 107 (15), 91 (100), 77 (9), 72 (22), 65 (36), 62 (2), 57 (11), 51 (4), 39 (12).

**Preparation of (***S***)-2-methylhex-5-en-1-ol (9).** Dilithium tetrachlorocuprate (0.1 M in THF, 2.46 ml, 0.246 mmol, 0.1 equiv) was added to a solution of tosylate **8** (600 mg, 2.46 mmol, 1.0 equiv) in degassed dry THF (25 ml) (Nunomoto et al. 1983). Allylmagnesium bromide (1 M in diethyl ether, 7.38 ml, 7.38 mmol, 3.0 equiv) was added dropwise over 20 min at 0 °C and the reaction mixture was stirred for 6 h at rt. Further allylmagnesium bromide (1 M in diethyl ether, 1.23 ml, 1.23 mmol, 0.5 equiv) was added dropwise to the reaction mixture at 0 °C. After stirring for 17 h at rt, the reaction mixture was quenched with sat. aq. NH_4_Cl solution (40 ml) and the phases were separated. The aq. layer was extracted with diethyl ether (3 × 40 ml), the comb. org. layers were washed with sat. aq. NH_4_Cl solution (100 ml), dried over anhydrous MgSO_4_, filtered and concentrated under reduce pressure. The residue was purified by column chromatography (silica gel, pentane/diethyl ether 1:1) to afford (*S*)-2-methylhex-5-en-1-ol (**9**) as a colorless liquid (268 mg, 2.35 mmol, 96%). R_*f*_ = 0.44 (pentane/diethyl ether 1:1); ${[\mathrm{\alpha }]}_{\mathrm{D}}^{25}$ = –12.3 (c = 1.00, DCM); ^1^H NMR (300 MHz, CDCl_3_) δ 5.82 (ddt, *J* = 16.8, 10.1, 6.6 Hz, 1H, CH), 5.09–4.91 (m, 2H, CH_2_), 3.52 (dd, *J* = 10.5, 5.8 Hz, 1H, C*H*_a_H_b_), 3.44 (dd, *J* = 10.5, 6.4 Hz, 1H, CH_a_*H*_b_), 2.23–1.96 (m, 2H, CH_2_), 1.79–1.39 (m, 2H, CH_2_), 1.44 (br. s, 1H, OH), 1.30–1.11 (m, 1H, CH), 0.93 (d, *J* = 6.7 Hz, 3H, CH_3_); ^13^C NMR (75 MHz, CDCl_3_) δ 138.9 (CH), 114.4 (CH_2_), 68.2 (CH_2_), 35.2 (CH), 32.3 (CH_2_), 31.2 (CH_2_), 16.4 (CH_3_); EIMS *m*/*z* (%) 114 (< 1) [M]^+^, 96 (11) [M–H_2_O]^+^, 82 (6), 81 (86), 79 (9), 71 (31), 68 (13), 67 (28), 58 (17), 57 (33), 56 (15), 55 (100), 54 (74), 53 (16), 51 (6), 45 (7), 43 (30), 42 (21), 41 (94), 40 (9), 39 (55).

**Preparation of (***S***)-2-methylhex-5-enoic acid (10).** Alcohol (*S*)-**9** (235 mg, 2.06 mmol, 1.0 equiv) was converted to carboxylic acid (*S*)-**10** using PDC (3.87 g, 10.3 mmol, 5.0 equiv) and DMF (10 ml) by following the general procedure B. The crude product was purified by column chromatography (silica gel, pentane/diethyl ether 4:1) to afford (*S*)-2-methylhex-5-enoic acid (**10**) as a colorless liquid (213 mg, 1.66 mmol, 81%). R_*f*_ = 0.62 (pentane/diethyl ether 1:1); ${[\mathrm{\alpha }]}_{\mathrm{D}}^{25}$ =  +24.8 (c = 1.00, DCM); ^1^H NMR (300 MHz, CDCl_3_) δ 10.59 (br. s, 1H, COOH), 5.79 (ddt, *J* = 16.9, 10.2, 6.6 Hz, 1H, CH), 5.09–4.95 (m, 2H, CH_2_), 2.50 (sext, *J* = 7.0 Hz, 1H, CH), 2.18–2.04 (m, 2H, CH_2_), 1.88–1.74 (m, 1H, C*H*_a_H_b_), 1.59–1.45 (m, 1H, CH_a_*H*_b_), 1.19 (d, *J* = 7.0 Hz, 3H, CH_3_); ^13^C NMR (75 MHz, CDCl_3_) δ 183.1 (C_q_), 137.7 (CH), 115.2 (CH_2_), 38.7 (CH), 32.5 (CH_2_), 31.2 (CH_2_), 16.8 (CH_3_); EIMS (TMS derivative) *m*/*z* (%) 200 (2) [M]^+^, 185 (10) [M–CH_3_]^+^, 146 (11), 143 (18), 131 (6), 75 (62), 74 (11), 73 (100), 56 (18), 55 (14), 47 (7), 45 (15), 43 (6), 41 (17), 39 (10).

**Preparation of (***R***)-3-((***tert***-butyldimethylsilyl)oxy)-2-methylpropyl 4-methylbenzenesulfonate (11).** Imidazole (274 mg, 4.02 mmol, 1.4 equiv), *tert*-butyldimethylsilyl chloride (TBSCl) (562 mg, 3.73 mmol, 1.3 equiv) and DMAP (17.6 mg, 0.144 mmol, 5 mol%) were added to a solution of alcohol **8** (700 mg, 2.87 mmol, 1.0 equiv) in dry THF (12 ml) at rt (Gieseler and Kalesse 2014). After stirring for 18 h at rt, the reaction mixture was diluted with pentane (10 ml) and sat. aq. NH_4_Cl solution (20 ml) was added. Water (5 ml) was added to improve phase separation. Then the phases were separated, and the aq. layer was extracted with pentane (3 × 20 ml). The comb. org. layers were washed with sat. aq. NaHCO_3_ solution (60 ml), dried over anhydrous MgSO_4_, filtered and concentrated under reduce pressure. The residue was purified by column chromatography (silica gel, pentane/diethyl ether 10:1) to afford (*R*)-3-((*tert*-butyldimethylsilyl)oxy)-2-methylpropyl 4-methylbenzenesulfonate (**11**) as a colorless oil (1.03 g, 2.87 mmol, quant.). R_*f*_ = 0.46 (pentane/diethyl ether 5:1); ${[\mathrm{\alpha }]}_{\mathrm{D}}^{25}$ = –5.7 (c = 1.00, DCM); ^1^H NMR (300 MHz, CDCl_3_) δ 7.81–7.76 (m, 2H, CH_Ar_), 7.36–7.31 (m, 2H, CH_Ar_), 4.02 (dd, *J* = 9.3, 5.9 Hz, 1H, C*H*_a_H_b_), 3.92 (dd, *J* = 9.3, 5.9 Hz, 1H, CH_a_*H*_b_), 3.50 (dd, *J* = 10.0, 5.0 Hz, 1H, C*H*_a_H_b_), 3.40 (dd, *J* = 10.0, 6.5 Hz, 1H, CH_a_*H*_b_), 2.46–2.43 (m, 3H, CH_3_), 2.03–1.86 (m, 1H, CH), 0.88 (d, *J* = 6.9 Hz, 3H, CH_3_), 0.82 (s, 9H, 3 × CH_3_), –0.02 (s, 6H, 2 × CH_3_); ^13^C NMR (75 MHz, CDCl_3_) δ 144.6 (C_q_), 133.1 (C_q_), 129.8 (2C, CH), 127.9 (2C, CH), 72.1 (CH_2_), 63.7 (CH_2_), 35.6 (CH), 25.8 (3C, CH_3_), 21.6 (CH_3_), 18.2 (C_q_), 13.2 (CH_3_), –5.58 (CH_3_), –5.61 (CH_3_); EIMS *m*/*z* (%) 271 (3), 231 (15), 230 (23), 229 (100), 165 (6), 155 (3), 149 (12), 131 (2), 115 (2), 101 (2), 91 (19), 85 (1), 75 (10), 73 (7), 65 (6), 59 (3), 57 (3), 55 (2), 41 (3).

**Preparation of 4-iodo-2-methylbut-1-ene (12).** Iodine (15.2 g, 59.9 mmol, 1.5 equiv) was added in portions at 0 °C to a solution of triphenylphosphine (15.7 g, 59.9 mmol, 1.5 equiv) and imidazole (5.43 g, 79.8 mmol, 2.0 equiv) in dry DCM (100 ml), followed by stirring for 15 min at this temperature (Helmboldt et al. 2006). Then 3-methylbut-3-en-1-ol (4.00 ml, 3.44 g, 39.9 mmol, 1.0 equiv) was added dropwise at 0 °C and the reaction mixture was stirred for 4.5 h at rt. The reaction mixture was quenched with sat. aq. sodium sulfite solution (80 ml), the phases were separated, and the aq. layer was extracted with DCM (2 × 80 ml). The comb. org. layers were washed with brine (200 ml), dried over anhydrous MgSO_4_, filtered, and concentrated under reduced pressure. The residue was filtered through a short column (Celite®, pentane) and purified by column chromatography (silica gel, pentane) to afford 4-iodo-2-methylbut-1-ene (**12**) as a colorless liquid (6.86 g, 35.0 mmol, 88%). R_*f*_ = 0.60 (pentane); ^1^H NMR (300 MHz, CDCl_3_) δ 4.88–4.84 (m, 1H, C*H*_a_H_b_), 4.77–4.74 (m, 1H, CH_a_*H*_b_), 3.26 (t, *J* = 7.6 Hz, 2H, CH_2_), 2.59 (t, *J* = 7.5 Hz, 2H, CH_2_), 1.76–1.72 (m, 3H, CH_3_); ^13^C NMR (75 MHz, CDCl_3_) δ 143.9 (C_q_), 112.3 (CH_2_), 41.9 (CH_2_), 21.7 (CH_3_), 3.50 (CH_2_).

**Preparation of (***S***)-***tert***-butyl((2,6-dimethylhept-6-en-1-yl)oxy)dimethylsilane (13).** The organolithium reagent was prepared from isoprenyl iodide (**12**, 1.28 g, 6.52 mmol, 9.5 equiv) and *t*-butyllithium (1.7 M in pentane, 7.65 ml, 13.0 mmol, 19.0 equiv, **Attention: careful handling required, extremely flammable reagent**) in a 3:2 mixture of dry and degassed hexane and diethyl ether (12.5 ml) by following the general procedure C. Then the coupling was performed with copper cyanide (292 mg, 3.26 mmol, 4.75 equiv) in dry diethyl ether (10 ml), organolithium solution and tosylate (*R*)-**11** (246 mg, 0.686 mmol, 1.0 equiv) in dry diethyl ether (5 ml) by following the general procedure C. The crude product was purified by column chromatography (silica gel, pentane) to afford (*S*)-*tert*-butyl((2,6-dimethylhept-6-en-1-yl)oxy)dimethylsilane (**13**) as a colorless liquid (148 mg, 0.577 mmol, 84%). R_*f*_ = 0.56 (pentane/diethyl ether 100:1); ${[\mathrm{\alpha }]}_{\mathrm{D}}^{25}$ = –3.6 (c = 1.00, DCM); ^1^H NMR (300 MHz, CDCl_3_) δ 4.71–4.64 (m, 2H, CH_2_), 3.45 (dd, *J* = 9.8, 5.9 Hz, 1H, C*H*_a_H_b_), 3.36 (dd, *J* = 9.8, 6.6 Hz, 1H, CH_a_*H*_b_), 1.99 (t, *J* = 7.4 Hz, 2H, CH_2_), 1.72–1.69 (m, 3H, CH_3_), 1.67–1.21 (m, 4H), 1.12–0.96 (m, 1H), 0.89 (s, 9H, 3 × CH_3_), 0.87 (d, *J* = 6.7 Hz, 3H, CH_3_), 0.04 (s, 6H, 2 × CH_3_); ^13^C NMR (75 MHz, CDCl_3_) δ 146.2 (C_q_), 109.7 (CH_2_), 68.4 (CH_2_), 38.1 (CH_2_), 35.7 (CH), 32.9 (CH_2_), 26.0 (3C, CH_3_), 25.0 (CH_2_), 22.4 (CH_3_), 18.4 (C_q_), 16.7 (CH_3_), –5.4 (2C, CH_3_); IR (neat) ṽ 2931 (m), 2893 (m), 2857 (m), 2323 (w), 1649 (w), 1464 (m), 1384 (w), 1367 (w), 1252 (m), 1091 (s), 1008 (w), 884 (m), 835 (s), 772 (s), 667 (m); EIMS *m*/*z* (%) 200 (3), 199 (16) [M–*t*Bu]^+^, 143 (4), 129 (2), 123 (2), 115 (7), 101 (3), 89 (6), 81 (4), 77 (4), 76 (8), 75 (100), 73 (15), 69 (5), 61 (3), 59 (7), 55 (7), 47 (3), 41 (11).

**Preparation of (***S***)-2,6-dimethylhept-6-en-1-ol (14).** Silyl ether (*S*)-**13** (394 mg, 1.54 mmol, 1.0 equiv) was converted to alcohol (*S*)-**14** using TBAF (1 M in THF/approx. 5% water, 1.85 ml, 1.85 mmol, 1.2 equiv) in dry THF (5 ml) by following the general procedure D. The crude product was purified by column chromatography (silica gel, pentane/diethyl ether 2:1) to afford (*S*)-2,6-dimethylhept-6-en-1-ol (**14**) as a colorless liquid (216 mg, 1.52 mmol, 99%). R_*f*_ = 0.37 (pentane/diethyl ether 2:1); ${[\mathrm{\alpha }]}_{\mathrm{D}}^{25}$ = –12.0 (c = 1.00, DCM); ^1^H NMR (300 MHz, CDCl_3_) δ 4.72–4.65 (m, 2H, CH_2_), 3.52 (dd, *J* = 10.5, 5.8 Hz, 1H, C*H*_a_H_b_), 3.43 (dd, *J* = 10.4, 6.5 Hz, 1H, CH_a_*H*_b_), 2.01 (t, *J* = 7.4 Hz, 2H, CH_2_), 1.73–1.70 (m, 3H, CH_3_), 1.73–1.30 (m, 4H), 1.33 (br. s, 1H, OH), 1.18–1.03 (m, 1H), 0.93 (d, *J* = 6.7 Hz, 3H, CH_3_); ^13^C NMR (75 MHz, CDCl_3_) δ 145.6 (C_q_), 109.4 (CH_2_), 67.9 (CH_2_), 37.6 (CH_2_), 35.3 (CH), 32.3 (CH_2_), 24.5 (CH_2_), 21.9 (CH_3_), 16.1 (CH_3_); IR (neat) ṽ 3334 (br), 3076 (w), 2929 (m), 2870 (m), 1648 (m), 1453 (m), 1375 (m), 1243 (w), 1103 (w), 1035 (s), 987 (m), 938 (w), 884 (s), 735 (m), 573 (m); EIMS *m*/*z* (%) 142 (4) [M]^+^, 124 (5) [M–H_2_O]^+^, 109 (45), 95 (29), 82 (59), 81 (37), 71 (23), 69 (75), 68 (74), 67 (65), 58 (14), 57 (29), 56 (74), 55 (76), 53 (24), 43 (25), 42 (11), 41 (100), 40 (10), 39 (48).

**Preparation of methyl (***S***)-3-((***tert***-butyldimethylsilyl)oxy)-2-methylpropanoate (16).** Imidazole (1.73 g, 25.4 mmol, 2.0 equiv), TBSCl (2.29 g, 15.2 mmol, 1.2 equiv) and DMAP (78.0 mg, 0.635 mmol, 5 mol%) were added at 0 °C to a solution of methyl (*S*)-3-hydroxy-2-methylpropanoate ((*S*)-Roche ester, **6**, 1.50 g, 12.7 mmol, 1.0 equiv) in dry DCM (13 ml) (Gieseler and Kalesse 2014). After stirring for 4 h at rt, the reaction mixture was diluted with DCM (10 ml) and sat. aq. NH_4_Cl solution (20 ml) was added. Water (5 ml) was added to improve phase separation. Then the phases were separated, and the aq. layer was extracted with DCM (2 × 20 ml). The comb. org. layers were dried over anhydrous MgSO_4_, filtered and concentrated under reduce pressure. The residue was purified by column chromatography (silica gel, pentane/diethyl ether 10:1) to afford methyl (*S*)-3-((*tert*-butyldimethylsilyl)oxy)-2-methylpropanoate (**16**) as a colorless liquid (2.31 g, 9.94 mmol, 78%). R_*f*_ = 0.54 (pentane/diethyl ether 10:1); ${[\mathrm{\alpha }]}_{\mathrm{D}}^{25}$ =  + 20.9 (c = 1.00, DCM); ^1^H NMR (300 MHz, CDCl_3_) δ 3.81–3.61 (m, 2H, CH_2_), 3.68 (s, 3H, CH_3_), 2.71–2.59 (m, 1H, CH), 1.14 (d, *J* = 7.0 Hz, 3H, CH_3_), 0.87 (s, 9H, 3 × CH_3_), 0.04 (d, *J* = 0.9 Hz, 6H, 2 × CH_3_); ^13^C NMR (75 MHz, CDCl_3_) δ 175.5 (C_q_), 65.2 (CH_2_), 51.5 (CH_3_), 42.5 (CH), 25.8 (3C, CH_3_), 18.2 (C_q_), 13.4 (CH_3_), –5.5 (2C, CH_3_); EIMS *m*/*z* (%) = 217 (3) [M–CH_3_]^+^, 201 (6), 176 (10), 175 (68) [M–*t*Bu]^+^, 147 (7), 119 (46), 105 (5), 91 (7), 90 (11), 89 (100), 75 (27), 73 (27), 59 (30), 58 (7), 57 (7), 45 (8), 41 (11).

**Preparation of (***R***)-3-((***tert***-butyldimethylsilyl)oxy)-2-methylpropan-1-ol (17).** Ester **16** (1.17 g, 5.03 mmol, 1.0 equiv) was reduced to alcohol **17** using sodium borohydride (420 mg, 11.1 mmol, 2.2 equiv) and boron trifluoride diethyl etherate (0.19 ml, 214 mg, 1.51 mmol, 0.3 equiv) in dry and degassed THF (14 ml) by following the general procedure A. The reaction was stirred for 24 h at rt. The crude product was purified by column chromatography (silica gel, pentane/diethyl ether 2:1) to afford (*R*)-3-((*tert*-butyldimethylsilyl)oxy)-2-methylpropan-1-ol (**17**) as a colorless liquid (715 mg, 3.50 mmol, 70%). R_*f*_ = 0.42 (pentane/diethyl ether 2:1); ${[\mathrm{\alpha }]}_{\mathrm{D}}^{25}$ =  + 10.0 (c = 1.00, DCM); ^1^H NMR (300 MHz, CDCl_3_) δ 3.77–3.71 (m, 1H, C*H*_a_H_b_), 3.68–3.51 (m, 3H, CH_a_*H*_b_, CH_2_), 2.76 (br. s, 1H, OH), 2.02–1.86 (m, 1H, CH), 0.90 (s, 9H, 3 × CH_3_), 0.84 (d, *J* = 7.0 Hz, 3H, CH_3_), 0.08 (s, 6H, 2 × CH_3_); ^13^C NMR (75 MHz, CDCl_3_) δ 68.8 (CH_2_), 68.3 (CH_2_), 37.0 (CH), 25.8 (3C, CH_3_), 18.2 (C_q_), 13.1 (CH_3_), –5.56 (CH_3_), –5.62 (CH_3_); EIMS *m*/*z* (%) 147 (24) [M–*t*Bu]^+^, 129 (6), 105 (58), 89 (6), 77 (12), 76 (16), 75 (100), 73 (26), 61 (6), 59 (12), 58 (6), 57 (6), 55 (13), 47 (9), 45 (12), 43 (6), 41 (10).

**Preparation of (***S***)-3-((***tert***-butyldimethylsilyl)oxy)-2-methylpropyl 4-methylbenzenesulfonate (11).** Triethylamine (0.65 ml, 475 mg, 4.69 mmol, 1.4 equiv), DMAP (61.5 mg, 0.503 mmol, 15 mol%) and tosyl chloride (831 mg, 4.36 mmol, 1.3 equiv) were added sequentially to a solution of alcohol **17** (685 mg, 3.35 mmol, 1.0 equiv) in dry DCM (5 ml) at 0 °C (Han 2018). After stirring for 18 h at rt, further triethylamine (0.09 ml, 67.8 mg, 0.670 mmol, 0.2 equiv) and tosyl chloride (128 mg, 0.670 mmol, 0.2 equiv) were added at 0 °C. The reaction mixture was stirred for additional 3 h at rt. Then water (10 ml) was added, the phases were separated, and the aq. layer was extracted with diethyl ether (3 × 10 ml). The comb. org. layers were washed with brine (30 ml), dried over anhydrous MgSO_4_, filtered and concentrated under reduce pressure. The residue was purified by column chromatography (silica gel, pentane/diethyl ether 2:1) to afford (*S*)-3-((*tert*-butyldimethylsilyl)oxy)-2-methylpropyl 4-methylbenzenesulfonate (**11**) as a colorless oil (1.04 g, 2.90 mmol, 87%). R_*f*_ = 0.46 (pentane/diethyl ether 5:1); ${[\mathrm{\alpha }]}_{\mathrm{D}}^{25}$ =  +6.2 (c = 1.00, DCM); ^1^H NMR (300 MHz, CDCl_3_) δ 7.81–7.76 (m, 2H, CH_Ar_), 7.37–7.31 (m, 2H, CH_Ar_), 4.02 (dd, *J* = 9.3, 5.9 Hz, 1H, C*H*_a_H_b_), 3.92 (dd, *J* = 9.2, 5.9 Hz, 1H, CH_a_*H*_b_), 3.50 (dd, *J* = 10.0, 5.0 Hz, 1H, C*H*_a_H_b_), 3.40 (dd, *J* = 10.0, 6.5 Hz, 1H, CH_a_*H*_b_), 2.46–2.43 (m, 3H, CH_3_), 2.02–1.87 (m, 1H, CH), 0.88 (d, *J* = 6.9 Hz, 3H, CH_3_), 0.82 (s, 9H, 3 × CH_3_), –0.02 (s, 6H, 2 × CH_3_); ^13^C NMR (75 MHz, CDCl_3_) δ 144.6 (C_q_), 133.1 (C_q_), 129.8 (2C, CH), 127.9 (2C, CH), 72.1 (CH_2_), 63.7 (CH_2_), 35.6 (CH), 25.8 (3C, CH_3_), 21.6 (CH_3_), 18.2 (C_q_), 13.2 (CH_3_), –5.58 (CH_3_), –5.61 (CH_3_); EIMS *m*/*z* (%) 271 (2), 231 (15), 230 (24), 229 (100), 165 (7), 155 (4), 149 (17), 91 (32), 77 (4), 75 (22), 73 (18), 65 (11), 59 (7), 57 (6), 55 (6), 41 (5).

**Preparation of (***R***)-***tert***-butyldimethyl((2-methylhex-5-en-1-yl)oxy)silane (18).** Dilithium tetrachlorocuprate (0.1 M in THF, 2.22 ml, 0.222 mmol, 0.1 equiv) was added to a solution of tosylate (*S*)-**11** (797 mg, 2.22 mmol, 1.0 equiv) in degassed and dry THF (22 ml) (Nunomoto et al. 1983). Allylmagnesium bromide (1 M in diethyl ether, 7.77 ml, 7.77 mmol, 3.5 equiv) was added dropwise over 15 min at 0 °C. The reaction mixture was stirred for 22 h at rt. Further allylmagnesium bromide (1 M in diethyl ether, 2.22 ml, 2.22 mmol, 1.0 equiv) was added dropwise to the reaction mixture at 0 °C. After stirring for 8 h at rt, the reaction mixture was quenched with sat. aq. NH_4_Cl solution (40 ml) and the phases were separated. The aq. layer was extracted with diethyl ether (3 × 40 ml). The comb. org. layers were washed with sat. aq. NH_4_Cl solution (100 ml), dried over anhydrous MgSO_4_, filtered and concentrated under reduce pressure. The residue was purified by column chromatography (silica gel, pentane/diethyl ether 100:1) to afford (*R*)-*tert*-butyldimethyl((2-methylhex-5-en-1-yl)oxy)silane (**18**) as a colorless liquid (324 mg, 1.42 mmol, 64%). R_*f*_ = 0.56 (pentane/diethyl ether 100:1); ${[\mathrm{\alpha }]}_{\mathrm{D}}^{25}$ =  +2.2 (c = 0.99, DCM); ^1^H NMR (300 MHz, CDCl_3_) δ 5.81 (ddt, *J* = 16.9, 10.2, 6.6 Hz, 1H, CH), 5.04–4.90 (m, 2H, CH_2_), 3.45 (dd, *J* = 9.8, 5.8 Hz, 1H, C*H*_a_H_b_), 3.38 (dd, *J* = 9.8, 6.3 Hz, 1H, CH_a_*H*_b_), 2.19–1.95 (m, 2H, CH_2_), 1.69–1.43 (m, 1H, CH), 1.37–1.07 (m, 2H, CH_2_), 0.89 (s, 9H, 3 × CH_3_), 0.88 (d, *J* = 6.6 Hz, 3H, CH_3_), 0.04 (s, 6H, 2 × CH_3_); ^13^C NMR (75 MHz, CDCl_3_) δ 139.3 (CH), 114.1 (CH_2_), 68.2 (CH_2_), 35.2 (CH), 32.4 (CH_2_), 31.3 (CH_2_), 26.0 (3C, CH_3_), 18.4 (C_q_), 16.6 (CH_3_), –5.4 (2C, CH_3_); IR (neat) ṽ 2929 (m), 2858 (m), 1640 (w), 1465 (m), 1390 (w), 1253 (m), 1094 (m), 1003 (w), 909 (m), 842 (s), 775 (m), 670 (m), 557 (m); EIMS *m*/*z* (%) 172 (3), 171 (18) [M–*t*Bu]^+^, 141 (3), 115 (10), 99 (3), 89 (5), 77 (4), 76 (7), 75 (100), 73 (19), 61 (3), 59 (8), 58 (4), 57 (4), 55 (7), 47 (4), 45 (4), 41 (9), 39 (4).

**Preparation of (***R***)-2-methylhex-5-en-1-ol (9).** Silyl ether (*R*)-**18** (300 mg, 1.31 mmol, 1.0 equiv) was converted to alcohol (*R*)-**9** using TBAF (1 M in THF/approx. 5% water, 1.57 ml, 1.57 mmol, 1.2 equiv) in dry THF (4 ml) by following the general procedure D. The crude product was purified by column chromatography (silica gel, pentane/diethyl ether 5:1) to afford (*R*)-2-methylhex-5-en-1-ol (**9**) as a colorless liquid (132 mg, 1.16 mmol, 89%). R_*f*_ = 0.44 (pentane/diethyl ether 5:1); ${[\mathrm{\alpha }]}_{\mathrm{D}}^{25}$ =  +11.3 (c = 1.01, DCM); ^1^H NMR (300 MHz, CDCl_3_) δ 5.82 (ddt, *J* = 16.9, 10.2, 6.6 Hz, 1H, CH), 5.06–4.92 (m, 2H, CH_2_), 3.52 (dd, *J* = 10.5, 5.8 Hz, 1H, C*H*_a_H_b_), 3.44 (dd, *J* = 10.5, 6.4 Hz, 1H, CH_a_*H*_b_), 2.22–1.97 (m, 2H, CH_2_), 1.74–1.40 (m, 2H, CH_2_), 1.45 (br. s, 1H, OH), 1.29–1.14 (m, 1H, CH), 0.93 (d, *J* = 6.7 Hz, 3H, CH_3_); ^13^C NMR (75 MHz, CDCl_3_) δ 138.9 (CH), 114.4 (CH_2_), 68.1 (CH_2_), 35.2 (CH), 32.3 (CH_2_), 31.2 (CH_2_), 16.4 (CH_3_); EIMS *m*/*z* (%) 114 (< 1) [M]^+^, 96 (12) [M–H_2_O]^+^, 82 (6), 81 (88), 79 (9), 71 (32), 68 (13), 67 (29), 58 (17), 57 (34), 56 (16), 55 (100), 54 (74), 53 (16), 51 (5), 45 (6), 43 (28), 42 (20), 41 (86), 40 (8), 39 (48).

**Preparation of (***R***)-2-methylhex-5-enoic acid (10).** Alcohol (*R*)-**9** (107 mg, 0.937 mmol, 1.0 equiv) was converted to carboxylic acid (*R*)-**10** using PDC (1.76 g, 4.69 mmol, 5.0 equiv) and DMF (5 ml) by following the general procedure B. The crude product was purified by column chromatography (silica gel, pentane/diethyl ether 4:1) to afford (*R*)-2-methylhex-5-enoic acid (**10**) as a colorless liquid (97.0 mg, 0.757 mmol, 81%). R_*f*_ = 0.62 (pentane/diethyl ether 1:1); ${[\mathrm{\alpha }]}_{\mathrm{D}}^{25}$ = –24.1 (c = 1.00, DCM); ^1^H NMR (300 MHz, CDCl_3_) δ 11.63 (br. s, 1H, COOH), 5.79 (ddt, *J* = 16.9, 10.2, 6.6 Hz, 1H, CH), 5.09–4.95 (m, 2H, CH_2_), 2.50 (sext, *J* = 7.0 Hz, 1H, CH), 2.17–2.06 (m, 2H, CH_2_), 1.88–1.75 (m, 1H, C*H*_a_H_b_), 1.59–1.46 (m, 1H, CH_a_*H*_b_), 1.20 (d, *J* = 7.0 Hz, 3H, CH_3_); ^13^C NMR (75 MHz, CDCl_3_) δ 183.3 (C_q_), 137.7 (CH), 115.2 (CH_2_), 38.7 (CH), 32.5 (CH_2_), 31.2 (CH_2_), 16.7 (CH_3_); EIMS (TMS derivative) *m*/*z* (%) 200 (4) [M]^+^, 185 (13) [M–CH_3_]^+^, 146 (15), 143 (23), 131 (8), 130 (14), 75 (66), 74 (11), 73 (100), 56 (18), 55 (14), 47 (7), 45 (15), 41 (16), 39 (9).

**Preparation of (***R*)-*tert***-butyl((2,6-dimethylhept-6-en-1-yl)oxy)dimethylsilane (13).** The organolithium reagent was prepared from isoprenyl iodide (**12**, 2.76 g, 14.1 mmol, 7.0 equiv) and *t*-butyllithium (**Attention: careful handling required, extremely flammable reagent,** 1.7 M in pentane, 16.5 ml, 28.1 mmol, 14.0 equiv) in a 3:2 mixture of dry and degassed hexane and diethyl ether (28 ml) by following the general procedure C. Then the coupling was performed with copper cyanide (631 mg, 7.04 mmol, 3.5 equiv) in dry diethyl ether (25 ml), organolithium solution and tosylate (*S*)-**11** (720 mg, 2.01 mmol, 1.0 equiv) in dry diethyl ether (14 ml) by following the general procedure C. The crude product was purified by column chromatography (silica gel, pentane) to afford (*R*)-*tert*-butyl((2,6-dimethylhept-6-en-1-yl)oxy)dimethylsilane (**13**) as a colorless liquid (439 mg, 1.71 mmol, 85%). R_*f*_ = 0.56 (pentane/diethyl ether 100:1); ${[\mathrm{\alpha }]}_{\mathrm{D}}^{25}$ =  +3.6 (c = 1.00, DCM); ^1^H NMR (300 MHz, CDCl_3_) δ 4.71–4.64 (m, 2H, CH_2_), 3.45 (dd, *J* = 9.8, 5.9 Hz, 1H, C*H*_a_H_b_), 3.36 (dd, *J* = 9.8, 6.6 Hz, 1H, CH_a_*H*_b_), 1.99 (t, *J* = 7.4 Hz, 2H, CH_2_), 1.73–1.69 (m, 3H, CH_3_), 1.67–1.20 (m, 4H), 1.13–0.96 (m, 1H), 0.89 (s, 9H, 3 × CH_3_), 0.87 (d, *J* = 6.7 Hz, 3H, CH_3_), 0.03 (s, 6H, 2 × CH_3_); ^13^C NMR (75 MHz, CDCl_3_) δ 146.2 (C_q_), 109.7 (CH_2_), 68.4 (CH_2_), 38.1 (CH_2_), 35.7 (CH), 32.9 (CH_2_), 26.0 (3C, CH_3_), 25.0 (CH_2_), 22.4 (CH_3_), 18.4 (C_q_), 16.7 (CH_3_), –5.4 (2C, CH_3_); EIMS *m*/*z* (%) 200 (6), 199 (35) [M–*t*Bu]^+^, 143 (7), 129 (4), 123 (4), 115 (12), 101 (4), 89 (9), 81 (6), 77 (6), 76 (10), 75 (100), 73 (20), 69 (7), 61 (4), 59 (8), 55 (9), 41 (13), 39 (4).

**Preparation of (***R***)-2,6-dimethylhept-6-en-1-ol (14).** Silyl ether (*R*)-**13** (398 mg, 1.55 mmol, 1.0 equiv) was converted to alcohol (*R*)-**14** using TBAF (1 M in THF/approx. 5% water, 1.86 ml, 1.86 mmol, 1.2 equiv) in dry THF (5 ml) by following the general procedure D. The crude product was purified by column chromatography (silica gel, pentane/diethyl ether 2:1) to afford (*R*)-2,6-dimethylhept-6-en-1-ol (**14**) as a colorless liquid (205 mg, 1.44 mmol, 93%). R_*f*_ = 0.37 (pentane/diethyl ether 2:1); ${[\mathrm{\alpha }]}_{\mathrm{D}}^{25}$ =  +12.1 (c = 1.00, DCM); ^1^H NMR (300 MHz, CDCl_3_) δ 4.72–4.65 (m, 2H, CH_2_), 3.51 (dd, *J* = 10.5, 5.8 Hz, 1H, C*H*_a_H_b_), 3.42 (dd, *J* = 10.5, 6.5 Hz, 1H, CH_a_*H*_b_), 2.01 (t, *J* = 7.4 Hz, 2H, CH_2_), 1.73–1.69 (m, 3H, CH_3_), 1.73–1.33 (m, 5H), 1.18–1.03 (m, 1H), 0.93 (d, *J* = 6.7 Hz, 3H, CH_3_); ^13^C NMR (75 MHz, CDCl_3_) δ 146.0 (C_q_), 109.8 (CH_2_), 68.3 (CH_2_), 38.0 (CH_2_), 35.7 (CH), 32.7 (CH_2_), 24.9 (CH_2_), 22.3 (CH_3_), 16.5 (CH_3_); EIMS *m*/*z* (%) 142 (3) [M]^+^, 124 (3) [M–H_2_O]^+^, 109 (30), 95 (23), 82 (51), 81 (32), 71 (23), 69 (72), 68 (73), 67 (59), 58 (15), 57 (30), 56 (81), 55 (77), 53 (21), 43 (28), 42 (11), 41 (100), 40 (9), 39 (41).

**Preparation of (***S***)-2,6-dimethylhept-6-en-1-yl (***S***)-2-methylhex-5-enoate (15).** The alcohol (*S*)-**14** (75.0 mg, 0.527 mmol, 1.0 equiv) and carboxylic acid (*S*)-**10** (67.0 mg, 0.527 mmol, 1.0 equiv) were esterified by following the general procedure E using EDC-HCl (111 mg, 0.580 mmol, 1.1 equiv) and DMAP (6.4 mg, 0.0527 mmol, 10 mol%) in dry DCM (2.5 ml) to yield (*S*)-2,6-dimethylhept-6-en-1-yl (*S*)-2-methylhex-5-enoate ((*S*,*S*)-**15**) as a colorless liquid (122 mg, 0.483 mmol, 92%). R_*f*_ = 0.48 (pentane/diethyl ether 20:1); ${[\mathrm{\alpha }]}_{\mathrm{D}}^{25}$ =  +11.5 (c = 1.00, DCM); ^1^H NMR (300 MHz, CDCl_3_) δ 5.79 (ddt, *J* = 16.9, 10.2, 6.6 Hz, 1H, CH), 5.06–4.93 (m, 2H, 2 × C*H*_a_H_b_), 4.72–4.64 (m, 2H, 2 × CH_a_*H*_b_), 3.96 (dd, *J* = 10.7, 5.9 Hz, 1H, C*H*_a_H_b_), 3.87 (dd, *J* = 10.7, 6.7 Hz, 1H, CH_a_*H*_b_), 2.47 (sext, *J* = 7.0 Hz, 1H, CH), 2.12–1.94 (m, 4H), 1.88–1.69 (m, 2H), 1.72–1.69 (m, 3H, CH_3_), 1.58–1.31 (m, 4H), 1.22–1.08 (m, 1H), 1.16 (d, *J* = 7.0 Hz, 3H, CH_3_), 0.94 (d, *J* = 6.7 Hz, 3H, CH_3_); ^13^C NMR (75 MHz, CDCl_3_) δ 176.7 (C_q_), 145.8 (C_q_), 137.9 (CH), 115.0 (CH_2_), 109.9 (CH_2_), 69.1 (CH_2_), 39.0 (CH), 37.9 (CH_2_), 32.94 (CH_2_), 32.86 (CH_2_), 32.5 (CH), 31.4 (CH_2_), 24.7 (CH_2_), 22.3 (CH_3_), 17.1 (CH_3_), 16.9 (CH_3_); IR (neat) ṽ 3076 (w), 2969 (m), 2933 (m), 2323 (w), 1732 (s), 1645 (w), 1457 (m), 1377 (m), 1171 (s), 991 (m), 911 (m), 886 (s), 749 (w), 643 (w), 538 (m); EIMS *m*/*z* (%) 252 (< 1) [M]^+^, 141 (1), 124 (12), 111 (19), 109 (24), 95 (18), 83 (65), 82 (87), 81 (43), 74 (19), 69 (94), 68 (59), 67 (37), 57 (11), 56 (25), 55 (100), 53 (14), 43 (12), 42 (11), 41 (74), 39 (22).

**Preparation of (***R***)-2,6-dimethylhept-6-en-1-yl (***R***)-2-methylhex-5-enoate (15).** The alcohol (*R*)-**14** (39.0 mg, 0.273 mmol, 1.0 equiv) and carboxylic acid (*R*)-**10** (35.0 mg, 0.273 mmol, 1.0 equiv) were esterified by following the general procedure E using EDC-HCl (58.0 mg, 0.300 mmol, 1.1 equiv) and DMAP (3.3 mg, 0.0273 mmol, 10 mol%) in dry DCM (1.5 ml) to yield (*R*)-2,6-dimethylhept-6-en-1-yl (*R*)-2-methylhex-5-enoate ((*R*,*R*)-**15**) as a colorless liquid (58.0 mg, 0.230 mmol, 84%). ${[\mathrm{\alpha }]}_{\mathrm{D}}^{20}$ = –11.7 (c = 1.01, DCM). The other analytical data were identical to that of its enantiomer, (*S*,*S*)-**15**.

**Preparation of (***R***)-2,6-dimethylhept-6-en-1-yl (***S***)-2-methylhex-5-enoate (15).** The alcohol (*R*)-**14** (65.0 mg, 0.457 mmol, 1.0 equiv) and carboxylic acid (*S*)-**10** (59.0 mg, 0.457 mmol, 1.0 equiv) were esterified by following the general procedure E using EDC-HCl (96.0 mg, 0.503 mmol, 1.1 equiv) and DMAP (5.6 mg, 0.0457 mmol, 10 mol%) in dry DCM (2.3 ml) to yield (*R*)-2,6-dimethylhept-6-en-1-yl (*S*)-2-methylhex-5-enoate ((*R*,*S*)-**15**) as a colorless liquid (98.0 mg, 0.388 mmol, 85%). ${[\mathrm{\alpha }]}_{\mathrm{D}}^{25}$ =  +15.0 (c = 1.00, DCM); ^1^H NMR (300 MHz, CDCl_3_) δ 5.79 (ddt, *J* = 16.9, 10.2, 6.6 Hz, 1H, CH), 5.06–4.93 (m, 2H, 2 × C*H*_a_H_b_), 4.72–4.64 (m, 2H, 2 × CH_a_*H*_b_), 3.96 (dd, *J* = 10.7, 5.9 Hz, 1H, C*H*_a_H_b_), 3.87 (dd, *J* = 10.7, 6.7 Hz, 1H, CH_a_*H*_b_), 2.47 (sext, *J* = 7.0 Hz, 1H, CH), 2.12–1.95 (m, 4H), 1.87–1.69 (m, 2H), 1.72–1.69 (m, 3H, CH_3_), 1.58–1.31 (m, 4H), 1.22–1.08 (m, 1H), 1.16 (d, *J* = 7.0 Hz, 3H, CH_3_), 0.94 (d, *J* = 6.7 Hz, 3H, CH_3_); ^13^C NMR (75 MHz, CDCl_3_) δ 176.7 (C_q_), 145.8 (C_q_), 137.9 (CH), 115.0 (CH_2_), 109.9 (CH_2_), 69.1 (CH_2_), 39.0 (CH), 37.9 (CH_2_), 32.94 (CH_2_), 32.86 (CH_2_), 32.5 (CH), 31.4 (CH_2_), 24.7 (CH_2_), 22.3 (CH_3_), 17.1 (CH_3_), 16.9 (CH_3_); IR (neat) ṽ 3076 (w), 2969 (m), 2934 (m), 1732 (s), 1645 (m), 1377 (m), 1241 (m), 1171 (s), 1049 (m), 991 (m), 911 (m), 886 (s), 748 (w), 636 (w); EIMS *m*/*z* (%) 252 (< 1) [M]^+^, 141 (1), 124 (10), 111 (17), 109 (22), 95 (17), 83 (62), 82 (86), 81 (42), 74 (18), 69 (95), 68 (60), 67 (35), 57 (11), 56 (24), 55 (100), 53 (13), 43 (13), 42 (11), 41 (76), 39 (21).

**Preparation of (***S***)-2,6-dimethylhept-6-en-1-yl (***R***)-2-methylhex-5-enoate (15).** The alcohol (*S*)-**14** (39.0 mg, 0.273 mmol, 1.0 equiv) and carboxylic acid (*R*)-**10** (35.0 mg, 0.273 mmol, 1.0 equiv) were esterified by following the general procedure E using EDC-HCl (58.0 mg, 0.300 mmol, 1.1 equiv) and DMAP (3.3 mg, 0.0273 mmol, 10 mol%) in dry DCM (1.5 ml) to yield (*S*)-2,6-dimethylhept-6-en-1-yl (*R*)-2-methylhex-5-enoate ((*S*,*R*)-**15**) as a colorless liquid (47.0 mg, 0.186 mmol, 68%). ${[\mathrm{\alpha }]}_{\mathrm{D}}^{25}$ = –15.5 (c = 1.01, DCM). The other analytical data were identical to that of its enantiomer, (*R*,*S*)-**15**.

**Preparation of (**2*S*,5*E*,10*S***)-2,6,10-trimethyl-5-undecen-11-olide (3).** The ester (*S*,*S*)-**15** (40.0 mg, 0.158 mmol, 1.0 equiv) was cyclized by following the general procedure F using Grubbs II generation catalyst (13.4 mg, 0.0158 mmol, 10 mol%), tetrafluoro-*p*-benzoquinone (2.8 mg, 0.0158 mmol, 10 mol%) and hexafluorobenzene (0.18 ml, 294 mg, 1.58 mmol, 10 equiv) in dry and degassed toluene (200 ml) to yield a mixture of the *E*/*Z* isomers (30.0 mg, 0.134 mmol, 85%) in a ratio of 37:63. After final purification by column chromatography (silica gel-AgNO_3_, pentane/diethyl ether 50:1), (2*S*,5*E*,10*S*)-2,6,10-trimethyl-5-undecen-11-olide ((2*S*,10*S*)*-***3**) was obtained as a colorless liquid (5.2 mg, 0.0232 mmol, 15%) in a ratio of *E*/*Z* 86:14. R_*f*_ = 0.48 (pentane/diethyl ether 20:1); ${[\mathrm{\alpha }]}_{\mathrm{D}}^{25}$ (*E*/*Z* 86:14) =  +22.3 ± 2.08 (c = 0.22, DCM); ^1^H NMR (500 MHz, CDCl_3_) δ 5.01–4.95 (m, 1H, CH), 4.45 (dd, *J* = 10.4, 3.6 Hz, 1H, C*H*_a_H_b_), 3.13 (t, *J* = 10.9 Hz, 1H, CH_a_*H*_b_), 2.32–2.15 (m, 2H, CH, C*H*_a_H_b_), 2.13–1.86 (m, 5H), 1.64–1.52 (m, 3H), 1.54 (s, 3H, CH_3_), 1.47–1.38 (m, 1H, CH_a_*H*_b_), 1.14 (d, *J* = 7.1 Hz, 3H, CH_3_), 0.92–0.79 (m, 1H), 0.81 (d, *J* = 6.8 Hz, 3H, CH_3_); ^13^C NMR (125 MHz, CDCl_3_) δ 177.7 (C_q_), 133.9 (C_q_), 126.2 (CH), 69.5 (CH_2_), 40.1 (CH), 35.9 (CH_2_), 33.8 (CH_2_), 28.6 (CH_2_), 27.4 (CH), 26.9 (CH_2_), 20.0 (CH_2_), 18.9 (CH_3_), 15.2 (CH_3_), 14.4 (CH_3_); IR (neat) ṽ 2923 (s), 2855 (m), 1733 (s), 1455 (m), 1372 (m), 1255 (w), 1179 (s), 1150 (s), 1097 (w), 1027 (w), 992 (w), 849 (w), 805 (m), 757 (w); HRMS (EI +) *m*/*z* [C_14_H_24_O_2_]^+^ calculated: 224.17763, found: 224.17707; EIMS *m*/*z* (%) 224 (6) [M]^+^, 206 (1), 191 (2), 181 (2), 168 (4), 163 (2), 151 (5), 135 (7), 126 (47), 109 (50), 95 (67), 81 (77), 67 (77), 55 (72), 41 (100); gas chromatographic retention index (Hydrodex β-6TBDM) *I* = 1618, (HP-5 MS) *I* = 1582.

**Preparation of (**2*R*,5*E*,10*R***)-2,6,10-trimethyl-5-undecen-11-olide (3).** The ester (*R*,*R*)-**15** (35.0 mg, 0.139 mmol, 1.0 equiv) was cyclized by following the general procedure F using Grubbs II generation catalyst (11.8 mg, 0.0139 mmol, 10 mol%), tetrafluoro-*p*-benzoquinone (2.5 mg, 0.0139 mmol, 10 mol%) and hexafluorobenzene (0.16 ml, 259 mg, 1.39 mmol, 10 equiv) in dry and degassed toluene (175 ml) to yield a mixture of the *E*/*Z* isomers (23.8 mg, 0.106 mmol, 76%) in a ratio of 36:64. After final purification by column chromatography (silica gel-AgNO_3_, pentane/diethyl ether 50:1), (2*R*,5*E*,10*R*)-2,6,10-trimethyl-5-undecen-11-olide ((2*R*,10*R*)*-***3**) was obtained as a colorless liquid (2.2 mg, 0.0098 mmol, 7%) in a ratio of *E*/*Z* 85:15. ${[\mathrm{\alpha }]}_{\mathrm{D}}^{25}$ (*E*/*Z* 85:15) = –22.3 ± 1.76 (c = 0.26, DCM); gas chromatographic retention index (Hydrodex β-6TBDM) *I* = 1614, (HP-5 MS) *I* = 1582. The other analytical data were identical to that of its enantiomer, (2*S*,10*S*)-**3**.

**Preparation of (**2*S*,5*E*,10*R***)-2,6,10-trimethyl-5-undecen-11-olide (3).** The ester (*R*,*S*)-**15** (40.0 mg, 0.158 mmol, 1.0 equiv) was cyclized by following the general procedure F using Grubbs II generation catalyst (13.4 mg, 0.0158 mmol, 10 mol%), tetrafluoro-*p*-benzoquinone (2.8 mg, 0.0158 mmol, 10 mol%) and hexafluorobenzene (0.18 ml, 294 mg, 1.58 mmol, 10 equiv) in dry and degassed toluene (200 ml) to yield a mixture of the *E*/*Z* isomers (30.3 mg, 0.135 mmol, 85%) in a ratio of 30:70. After final purification by column chromatography (silica gel-AgNO_3_, pentane/diethyl ether 50:1), (2*S*,5*E*,10*R*)-2,6,10-trimethyl-5-undecen-11-olide ((2*S*,10*R*)*-***3**) was obtained as a colorless liquid (3.9 mg, 0.0174 mmol, 11%) in a ratio of *E*/*Z* 97:3. ${[\mathrm{\alpha }]}_{\mathrm{D}}^{25}$ (*E*/*Z* 97:3) = –45.5 ± 3.03 (c = 0.20, DCM); ^1^H NMR (500 MHz, CDCl_3_) δ 5.18–5.12 (m, 1H, CH), 4.39 (dd, *J* = 10.4, 3.6 Hz, 1H, C*H*_a_H_b_), 3.28 (t, *J* = 10.8 Hz, 1H, CH_a_*H*_b_), 2.52–2.43 (m, 1H, CH), 2.43–2.34 (m, 1H), 2.07–1.85 (m, 5H), 1.78–1.69 (m, 1H), 1.61–1.50 (m, 2H), 1.55 (s, 3H, CH_3_), 1.47–1.37 (m, 1H), 1.13 (d, *J* = 7.1 Hz, 3H, CH_3_), 0.92–0.80 (m, 1H), 0.82 (d, *J* = 6.8 Hz, 3H, CH_3_); ^13^C NMR (125 MHz, CDCl_3_) δ 176.9 (C_q_), 134.2 (C_q_), 125.7 (CH), 69.3 (CH_2_), 41.0 (CH), 35.9 (CH_2_), 34.3 (CH_2_), 28.5 (CH_2_), 27.8 (CH), 26.1 (CH_2_), 20.3 (CH_2_), 17.4 (CH_3_), 15.3 (CH_3_), 14.4 (CH_3_); IR (neat) ṽ 2930 (s), 2858 (m), 1726 (s), 1453 (m), 1378 (w), 1343 (w), 1260 (m), 1231 (m), 1182 (m), 1092 (w), 1070 (w), 1037 (m), 804 (w), 676 (w); EIMS *m*/*z* (%) 224 (7) [M]^+^, 206 (1), 191 (3), 181 (2), 168 (5), 163 (3), 151 (6), 135 (9), 126 (58), 109 (61), 95 (80), 81 (85), 67 (80), 55 (74), 41 (100); gas chromatographic retention index (Hydrodex β-6TBDM) *I* = 1624, (HP-5 MS) *I* = 1595.

**Preparation of (**2*R*,5*E*,10*S***)-2,6,10-trimethyl-5-undecen-11-olide (3).** The ester (*R*,*S*)-**15** (30.0 mg, 0.119 mmol, 1.0 equiv) was cyclized by following the general procedure F using Grubbs II generation catalyst (10.1 mg, 0.0119 mmol, 10 mol%), tetrafluoro-*p*-benzoquinone (2.1 mg, 0.0119 mmol, 10 mol%) and hexafluorobenzene (0.14 ml, 221 mg, 1.19 mmol, 10 equiv) in dry and degassed toluene (150 ml) to yield a mixture of the *E*/*Z* isomers (22.0 mg, 0.0981 mmol, 82%) in a ratio of *E*/*Z* 21:79. After final purification by column chromatography (silica gel-AgNO_3_, pentane/diethyl ether 50:1), (2*R*,5*E*,10*S*)-2,6,10-trimethyl-5-undecen-11-olide ((2*R*,10*S*)*-***3**) was obtained as a colorless liquid (1.8 mg, 0.0080 mmol, 7%) in a ratio of *E*/*Z* 88:12. ${[\mathrm{\alpha }]}_{\mathrm{D}}^{25}$ (*E*/*Z* 88:12) =  +10.5 ± 2.07 (c = 0.20, DCM); gas chromatographic retention index (Hydrodex β-6TBDM) *I* = 1629, (HP-5 MS) *I* = 1595. The other analytical data were identical to that of its enantiomer, (2*S*,10*R*)-**3**.

## Results and Discussion

GC/MS analysis of an extract of the gular gland of *Hyperolius cinnamomeoventris* showed the presence of a cadinol (**4**) derivative and unknown sesquiterpenes, as well as the three macrocyclic lactones cucujolide III (**5**) (Menke et al. 2016), frogolide (**2**) (Menke et al. 2018) as well as compound **A** (Fig. 2).

**Fig. 3:**
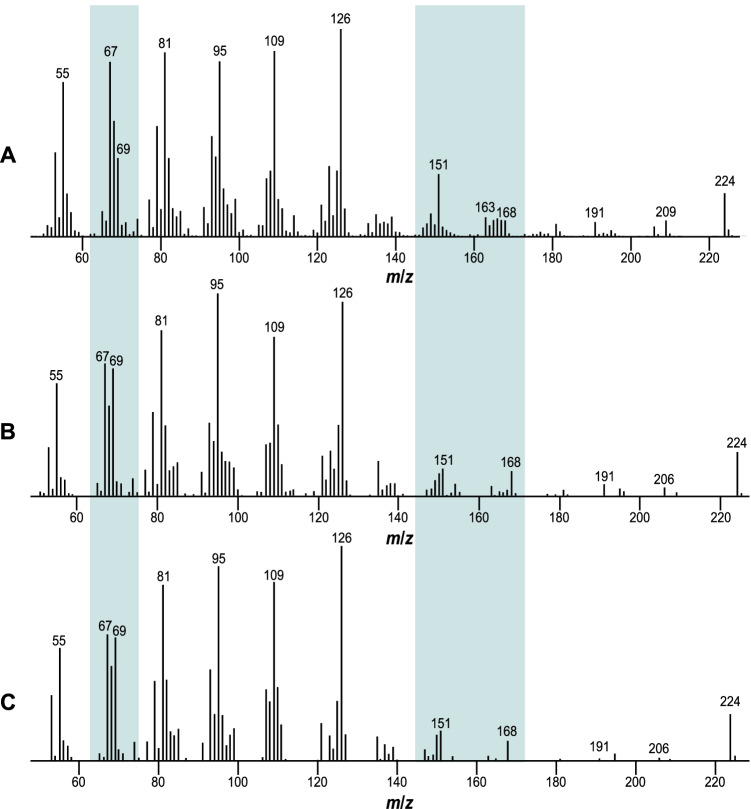
EI mass spectra of **A**: gephyromantolide A (**1**), **B**: compound **A** from *Hyperolius cinnamomeoventris*, and **C**: synthetic (2*R*,10*S*)-2,6,10-trimethyl-5-undecen-11-olide (**3**). The relevant differences are highlighted

At first glance, compound **A** exhibited a similar mass spectrum compared to that of gephyromantolide A (**1**), but there are significant differences (Fig. 3A and B). The ions at *m*/*z* 69 and 168 show higher intensities in the mass spectrum of compound **A**, while the ion *m*/*z* 151 is of higher intensity in **1**.

Compound **A** was therefore assumed to be an isomer of gephyromantolide A (**1**) in which the double bond is located at the generic terpene position, 2,6,10-trimethyl-5-undecen-11-olide (**3**). This position can be rationalized as follows. Although the biosynthetic origin of **1** has not been investigated, a terpenoid origin seems likely, with farnesyl pyrophosphate showing a 2,6,10-arrangement of double bonds being the precursor (Schulz et al. 2021). A loss of C-1 is needed during the biosynthetic formation of **1**, locating the remaining double bonds at C-5 and C-9. While the latter double bond is hydrogenated during the formation of gephyromantolide A, the C-5 double bond needs to be isomerized to C-6. If this isomerization does not take place, but all other steps remain identical, the proposed structure **3** would be formed. Since double bonds can be induced to migrate after hard mass spectrometric ionization at 70 eV, characteristic fragment ions indicating their original position are rarely observed. Therefore, double bond isomers often have very similar mass spectra and derivatization, or special mass spectrometric methods are required to localize double bonds (Buser et al. 1983; Jham et al. 2005; Ando and Yamakawa 2011; Kroiss et al. 2011). To determine the position of the double bond and their stereochemistry, a reference compound would be necessary to compare the mass spectra (Francke 2010). For this purpose, the double bond regioisomer of **1**, namely **3**, was synthesized stereoselectively.

**Fig. 4:**
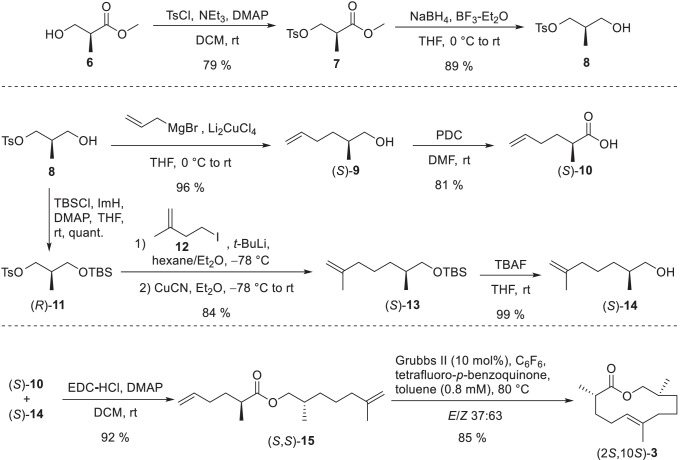
Synthesis of the (*S*)-configured building blocks (*S*)-**10** and (*S*)-**14** and the double bond isomer (2*S*,5*E*,10*S*)-2,6,10-trimethyl-5-undecen-11-olide (**3**)

**Fig. 5:**
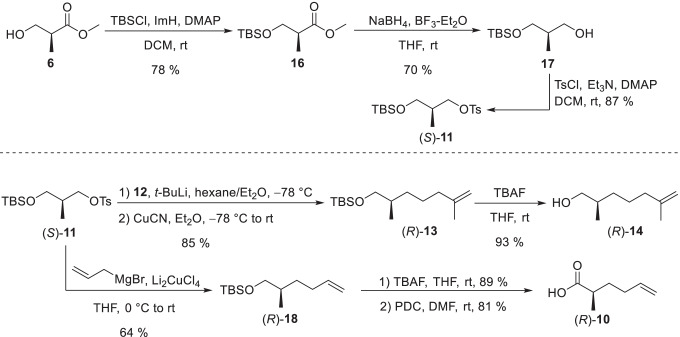
Synthesis of the (*R*)-building blocks (*R*)-**10** and (*R*)-**14**

The synthesis was planned to allow easy access of all stereoisomers for later determination of the absolute configuration (Fig. 4). Therefore, the internal double bond should be introduced by ring-closing-metathesis as (Schulz et al. 2021), allowing independent construction of two (*R*)- or (*S*)-configured building blocks. This allowed rapid access to all enantiomers by respective coupling to the individual ester precursor for olefin metathesis. The (*S*)-Roche ester (**6**) was selected as suitable enantiopure starting material.

(*S*)-Roche ester (**6**) was tosylated with tosyl chloride (Han 2018) followed by a reduction with sodium borohydride in presence of boron trifluoride (Zhang and Li 2018) to obtain alcohol **8**. A copper-mediated coupling with allylmagnesium bromide (Nunomoto et al. 1983) and subsequent oxidation with PDC (Peram et al. 2017) yielded the acid building block (*S*)-**10**. For the synthesis of the second building block, alcohol **8** was protected with a TBS group (Gieseler and Kalesse 2014). During the subsequent coupling, isoprenyl iodide (**12**) was converted into an organolithium reagent (Nurdin et al. 2020) and the respective Gilman cuprate was formed with copper cyanide (Chiou and Chen 2017). The coupling product (*S*)-**13** was deprotected with TBAF (Dash et al. 2015) leading to alcohol (*S*)-**14**. The TBS protection proved to be necessary because a direct coupling of tosylate **8** led to the racemization of the stereogenic center of **14**. During the reaction, **8** presumably cyclizes to the achiral 3-methyloxetane, which can be opened from both sides by the cuprate. Subsequent Steglich esterification (Chinta et al. 2016) of alcohol (*S*)-**14** with acid (*S*)-**10** gave ester (*S*,*S*)-**15**. Finally, ring-closing metathesis was performed using the Grubbs II catalyst (Peram et al. 2017) resulting in a difficult to separate *E*/*Z* mixture (ratio 37:63) of the target macrolide. The two stereoisomers were separated on silica gel impregnated with silver nitrate (Li et al. 1995) leading to (2*S*,5*E*,10*S*)-2,6,10-trimethyl-5-undecen-11-olide ((*S*,*S*)-**3**) in 15% yield. The (*R*)-building blocks (*R*)-**10** and (*R*)-**14** were also synthesized from (*S*)-Roche ester (**6**) in a similar way, thus needing only one enantiopure starting material to address all stereogenic centers (Fig. 5).

In an inverted reaction sequence, ester **6** was first protected with a TBS group followed by reduction with sodium borohydride to obtain alcohol **17**. A tosylation yielded the tosylate (*S*)-**11**. The (*R*)-configured alcohol building block (*R*)-**14** was synthesized by coupling tosylate (*S*)-**11** with isoprenyl iodide (**12**) (Chiou and Chen 2017; Nurdin et al. 2020) and subsequent deprotection with TBAF. Coupling of tosylate (*S*)-**11** with allylmagnesium bromide (Nunomoto et al. 1983) followed by deprotection with TBAF and oxidation with PDC led to the (*R*)-acid building block (*R*)-**10**. With all different building blocks now available, the three remaining stereoisomers of **3** were prepared analogously to (2*S*,10*S*)-**3**, namely (2*S*,10*R*)-**3**, (2*R*,10*S*)-**3**, and (2*R*,10*R*)-**3**.

A comparison of the mass spectra of compound **A** from *H. cinnamomeoventris* and the synthetic macrocyclic lactones showed good agreement between **A** and **3** (Fig. 3B, and C). The intensity of the ions *m*/*z* 69, 151, and 168 matched. Based on the mass spectra, compound **A** in the natural extract of *Hyperolius cinnamomeoventris* is the double bond isomer **3** of gephyromantolide A (**1**).

**Fig. 6:**
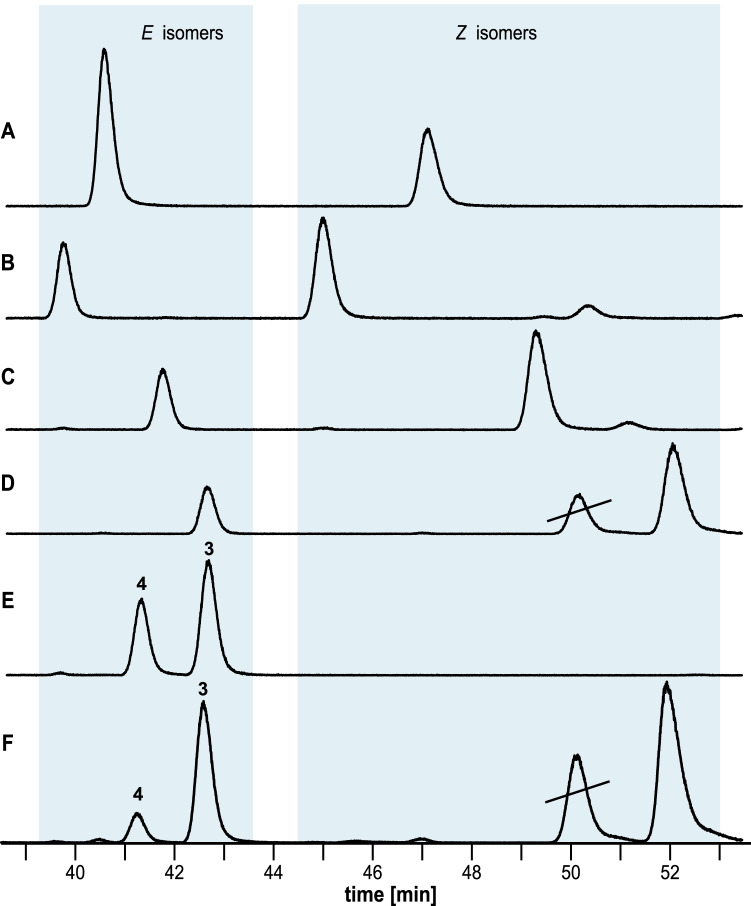
Determination of the absolute configuration of the macrocyclic lactone **3** from *Hyperolius cinnamomeoventris* by GC on a chiral Hydrodex β-6TBDM phase. **A**: (2*S*,10*S*)-**3**, **B**: (2*R*,10*R*)-**3**, **C**: (2*S*,10*R*)-**3**, **D**: (2*R*,10*S*)-**3**, **E**: natural lactone **A**, and **F**: coinjection of (2*R*,10*S*)-**3** and natural lactone **A**. Strike through denotes impurities

With the four stereoisomers in hand, the absolute configuration of the natural compound **A** was determined. This is important because chirality is a significant factor in the functionality of semiochemicals for species recognition (Mori 2007). For this purpose, the *E*/*Z* mixtures of all four stereoisomers were separated by GC on a chiral Hydrodex β-6TBDM phase (Fig. 6A–E). Since the peak of the (2*R*,5*E*,10*S*)-stereoisomer coincided with the naturally occurring lactone from *H. cinnamomeoventris*, a coinjection of the two samples was performed for confirmation (Fig. 6F). An intensification of the compound **A** peak was observed upon coinjection proved that the natural lactone is (2*R*,5*E*,10*S*)-2,6,10-trimethyl-5-undecen-11-olide. This compound proved to be a new natural product for which we propose the name cinnamomeoventrolide ((2*R*, 5*E*,10*S*)-**3**).

**Fig. 7:**
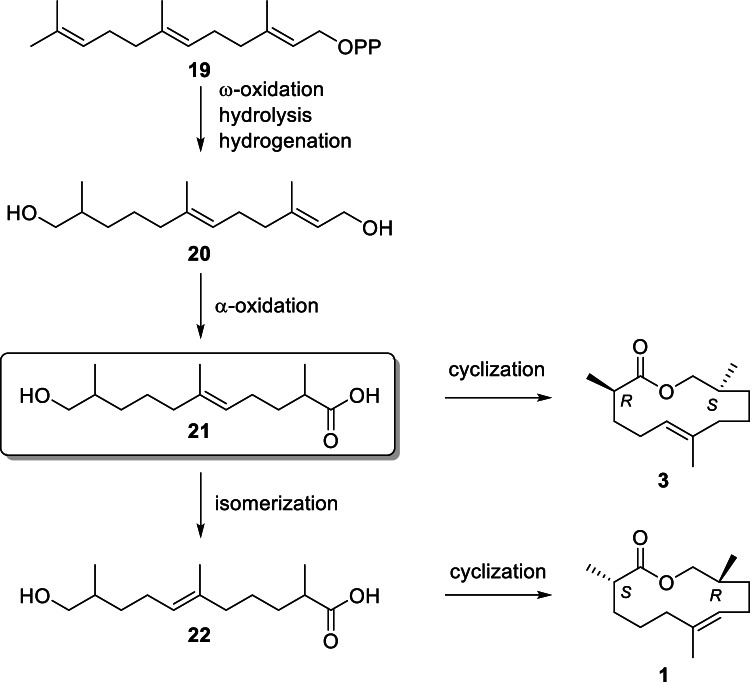
Possible biosynthesis of gephyromantolide A (**1**) and cinnamomeoventrolide (**3**). PP: pyrophosphate

A likely terpenoid biosynthetic pathway to both **1** and **3** is shown in Fig. 7. Farnesyl pyrophosphate (**19**) might be terminally oxidized and hydrogenated to form diol **20**. Subsequently, hydroxy acid **21** is formed by α-oxidation, losing one carbon via an acid intermediate. Hydroxy acid **22** is synthesized via isomerization of the double bond, followed by a final cyclization step to arrive at gephyromantolide A (**1**). Cinnamomeoventrolide (**3**) could be formed via cyclization of key intermediate hydroxy acid **21**, if the final isomerization does not take place.

The biosynthetic proposal indicates the close relationship between **1** and **3**. Interestingly, although both natural macrolides represent the respective (R*,S*)-diastereomer, they are opposite enantiomers. While gephyromantolide A (**1**) needs an additional biosynthetic isomerization step compared to cinnamomeoventrolide (**3**), the first steps in the biosynthesis are identical, although they are performed with opposite stereochemistry. This might indicate a significant difference between hyperolid and mantelline frogs. Earlier analyses have shown that *G. klemmeri* and *G. decaryi* also contain **1**, but not **3** (S. Schulz, unpublished), although we have so far not detected **3** in other hyperolids. Nevertheless, more species must be analyzed to see whether this difference is a consistent trait differentiating the families. The simple sesquiterpene lactone frogolide (**2**) occurs in both families, without differences in double bond positions (Menke et al. 2018). Cinnamomeoventrolide is only one major component of the gular secretion of *H. cinnamomeoventris*, as well as (*Z*)-tetradec-5-en-13-olide (**5**) (Menke et al. 2016), frogolide (**2**) and several sesquiterpenes including **4** (Menke et al. 2018). The exact function of this secretion in the hyperolids is still unknown, but the occurrence of the gland on the gular sac, the intense yellow belly of the males, and the enervation of the glands only during the mating season indicate a trimodal communication in these frogs, integrating acoustic, visual and chemical cues (Starnberger et al. 2013, 2014a, b). Because the secretion is species-specific, species recognition and attraction might be induced by cinnamomeoventrolide and other secretion components.

Interestingly, a similar switch between double bond positions in macrolides **1** and **3** is also occurring in insects. While (4*E*,8*E*)-4,8-dimethyldeca-4,8-dien-10-olide (ferrulactone I or cucujolide I) is a pheromone of the beetle *Cryptolestes ferrugineus* (Wong et al. 1983) and the butterfly *Pieris rapae* (Yildizhan et al. 2009), its isomer, (3*E*,8*E*)-4,8-dimethyldeca-3,8-dien-10-olide (suspensolide), is a pheromone component of *Anastrepha* fruit flies (Battiste et al. 1983; Chuman et al. 1988; Rocca et al. 1992). Macrolides are favored compounds for chemical communication, because non-volatile precursors are easily transferred into more volatile compounds (Schulz and Hötling 2015). Therefore, different lineages of frogs or insects have evolved to the same pheromone compounds, as in the case of phoracantholide J in beetles (Moore and Brown 1976) and frogs (Poth et al. 2012). Although it is known that poison frogs obtain their alkaloids from feeding on arthropods, we do not yet have evidence that this is the case of the macrolides observed as frog volatiles. Experiments with phoracantholides showed that mantellines can take-up macrolides with their diet, but are also able to synthesize them *de novo* (Schulz et al. 2021). Given the relatively small amounts of semiochemicals produced by insects, a diet uptake of macrolides by frogs cannot be excluded, but currently seems unlikely.

In summary, we have revealed for the first time the occurrence of structural regioisomers of anuran semiochemicals potentially used in species recognition and established the structure and the absolute configuration of, and synthetic access to a new terpenoid macrocyclic lactone from hyperolid frogs, cinnamomeoventrolide (**3**).

## Supplementary Information

Below is the link to the electronic supplementary material.

Supplementary file1 (PDF 1194 KB)
