# Supplementary material for: Cinnamomeoventrolide – Double Bond Regioisomerism in Frog Semiochemicals
Source: J Chem Ecol. 2022 Jul 9;48(5-6):531–45. doi: 10.1007/s10886-022-01370-6 (PMC9375755; doi:10.1007/s10886-022-01370-6)
Supplement: Supplementary file 1 — Supplementary file1 (PDF 1194 KB) [file 10886_2022_1370_MOESM1_ESM.pdf]

Supporting Information for the Journal of Chemical Ecology

CINNAMOMEOVENTROLIDE – DOUBLE BOND REGIOISOMERISM IN FROG SEMIOCHEMCIALS

JOHANNA KUHN, STEFAN SCHULZ*

Institute of Organic Chemistry, Technische Universität Braunschweig, Hagenring 30, 38106 Braunschweig, Germany

Corresponding author: Stefan Schulz, stefan.schulz@tu-braunschweig.de

ORCID Stefan Schulz: 0000-0002-4810-324X

Content

Mass spectra	2
NMR spectra of synthesized compounds	4

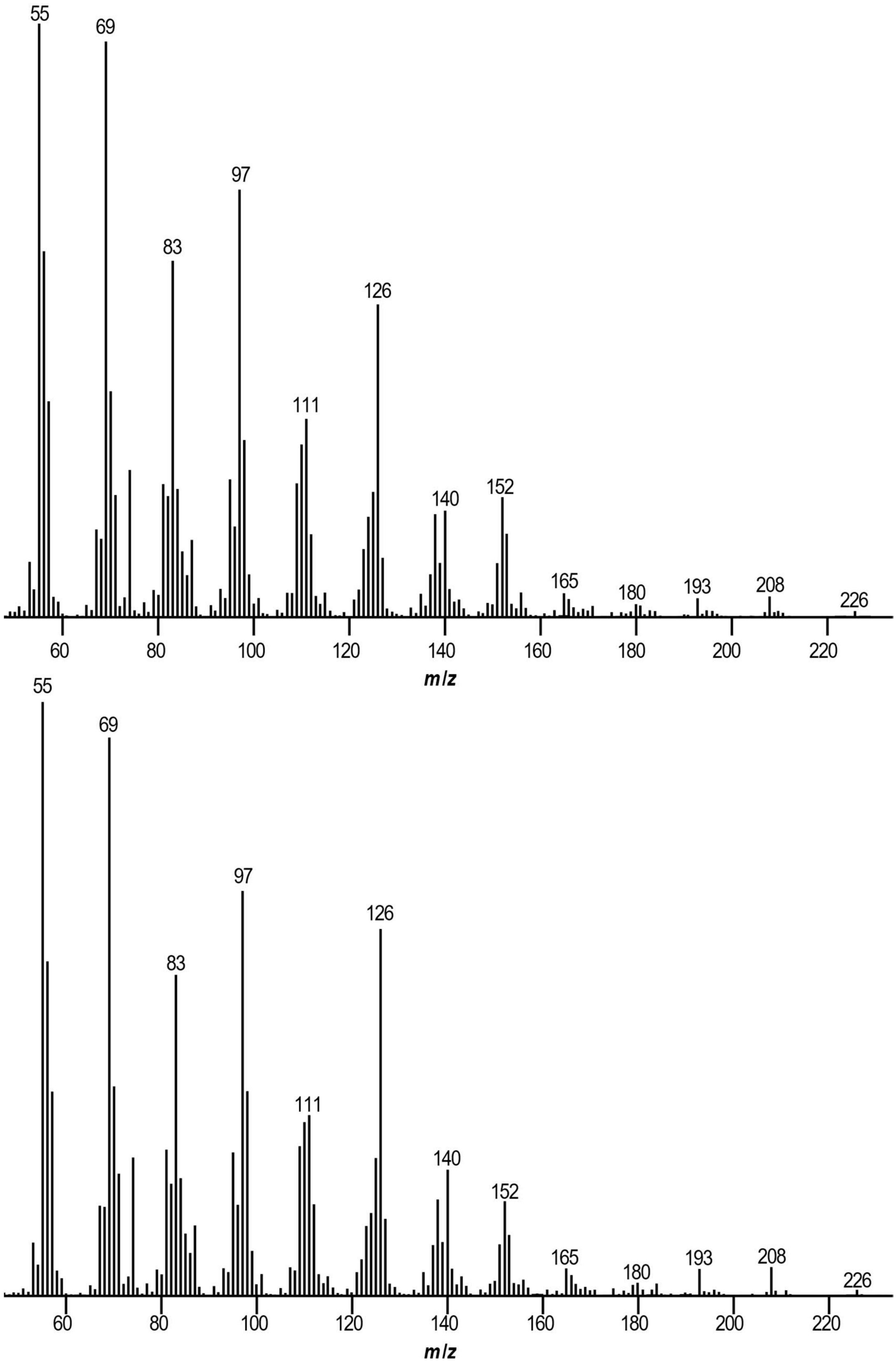

Figure S1: Mass spectra of hydrogenated cinnamomeoventrolide. Due to the stereogenic centers, two major diastereomers were foremed.

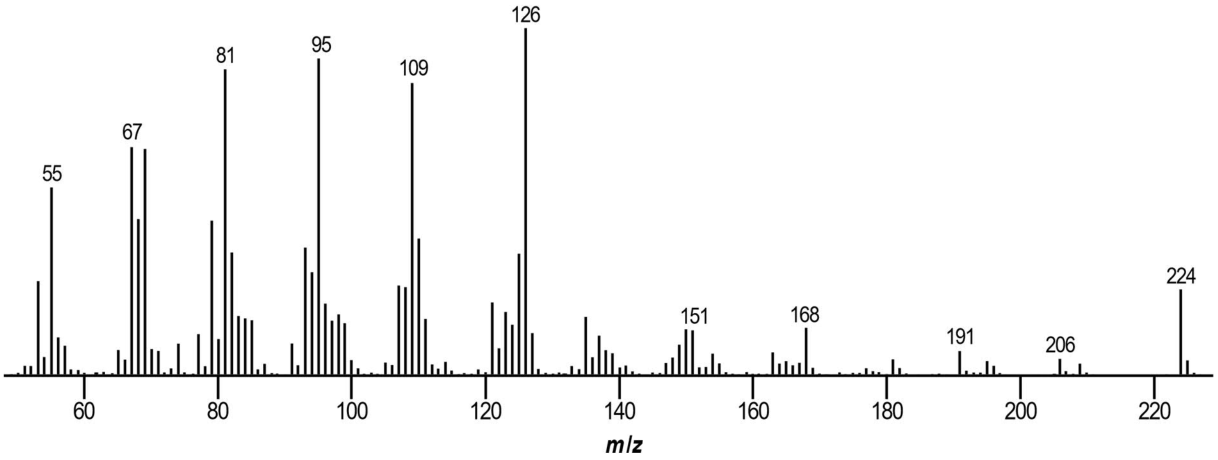

Figure S2: Mass spectrum of unnatural (2*S*,5*E*,10*S*)-2,6,10-trimethyl-5-undecen-11-olide (**3**).

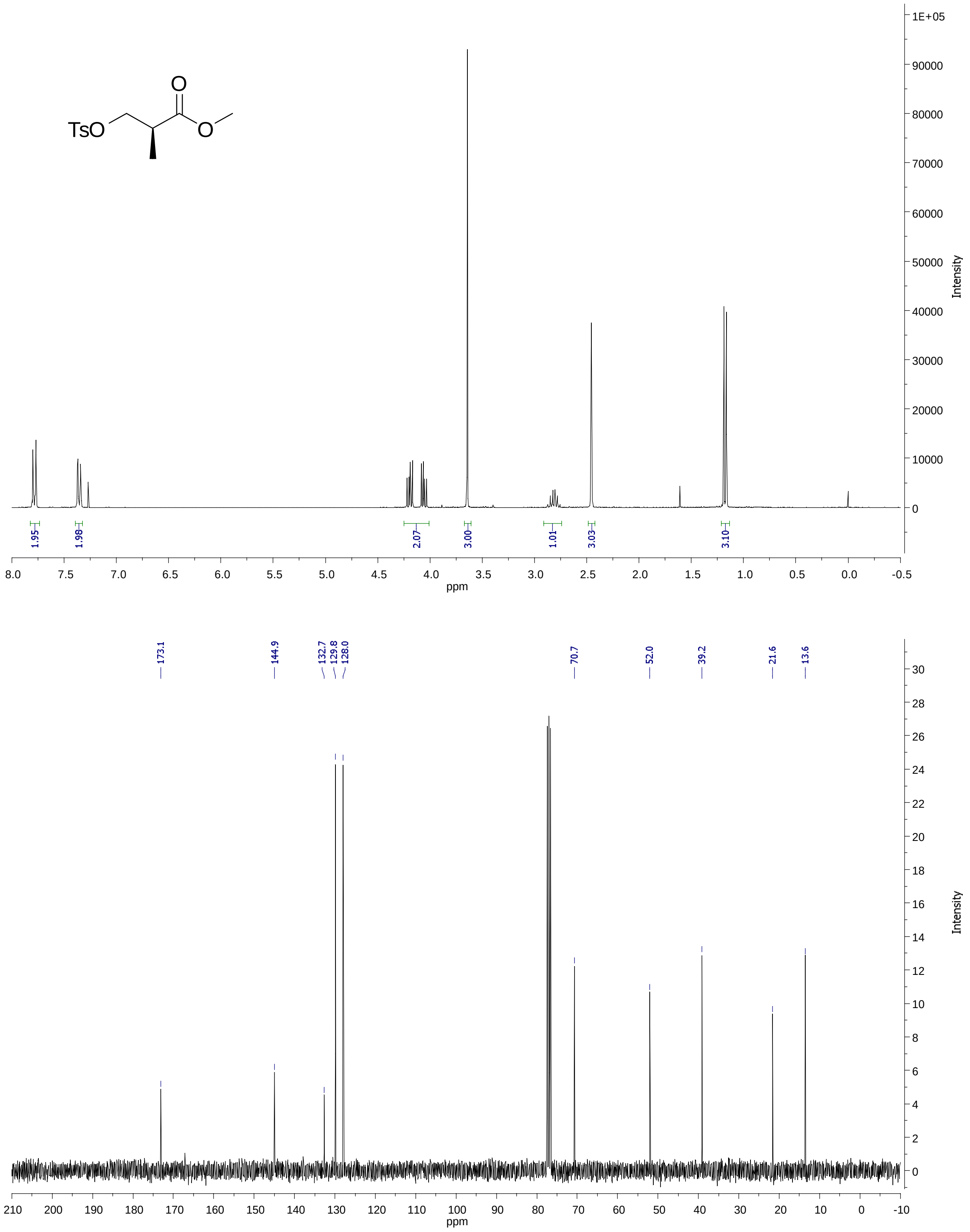

Figure S3: ^{1}H NMR (300 MHz, $CDCl_3$) and ^{13}C NMR (75 MHz, $CDCl_3$) spectrum of methyl (*S*)-2-methyl-3-(tosyloxy)propanoate (**7**).

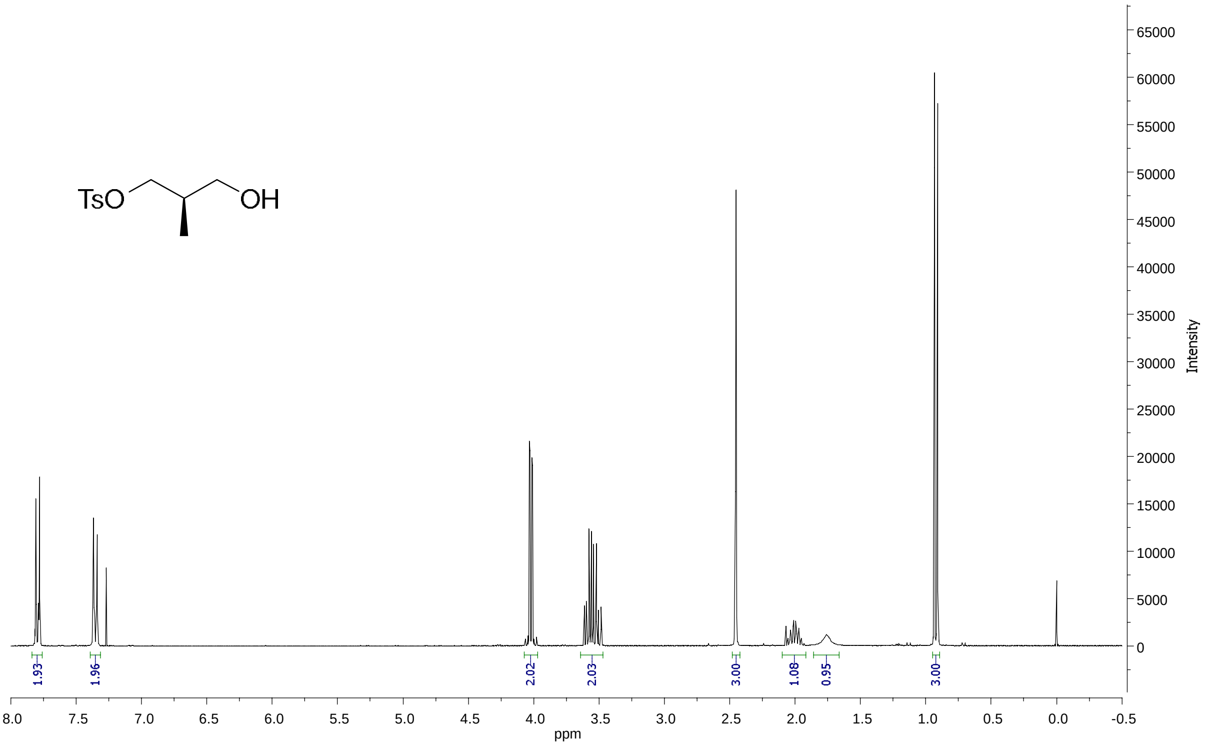

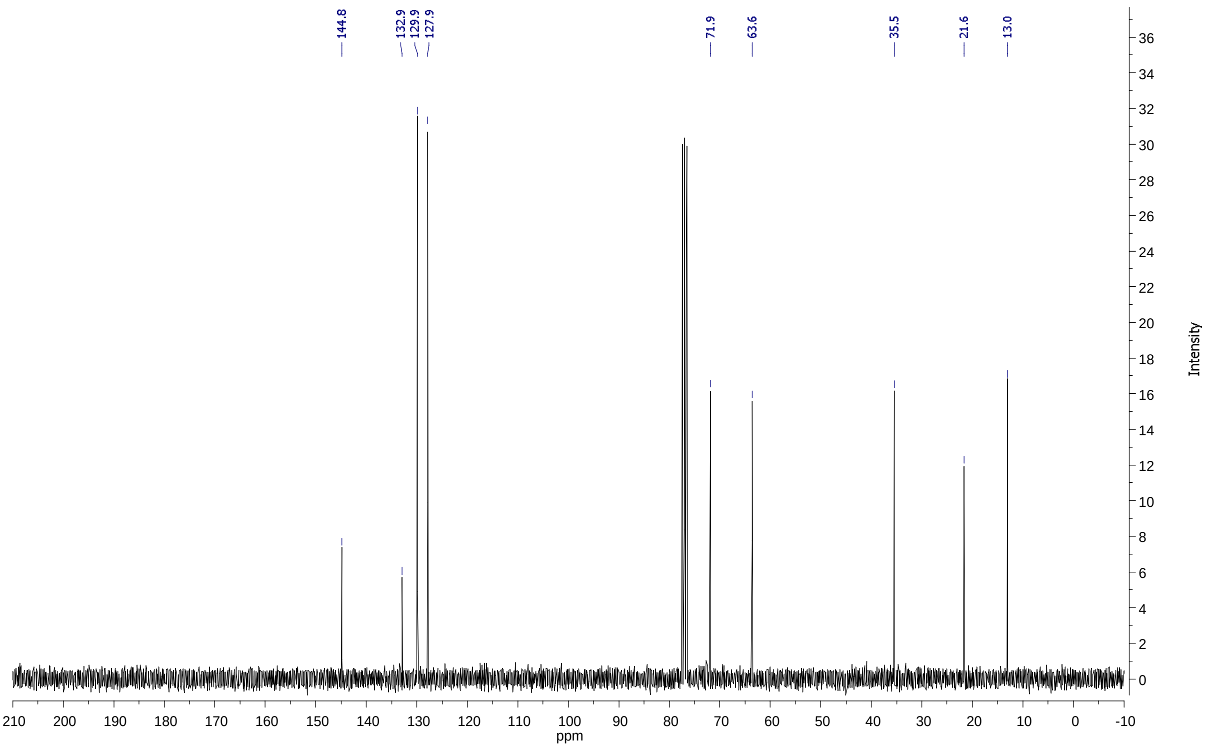

Figure S4: ^{1}H NMR (300 MHz, $CDCl_3$) and ^{13}C NMR (75 MHz, $CDCl_3$) spectrum of (*R*)-3-hydroxy-2-methylpropyl 4-methylbenzenesulfonate (**8**).

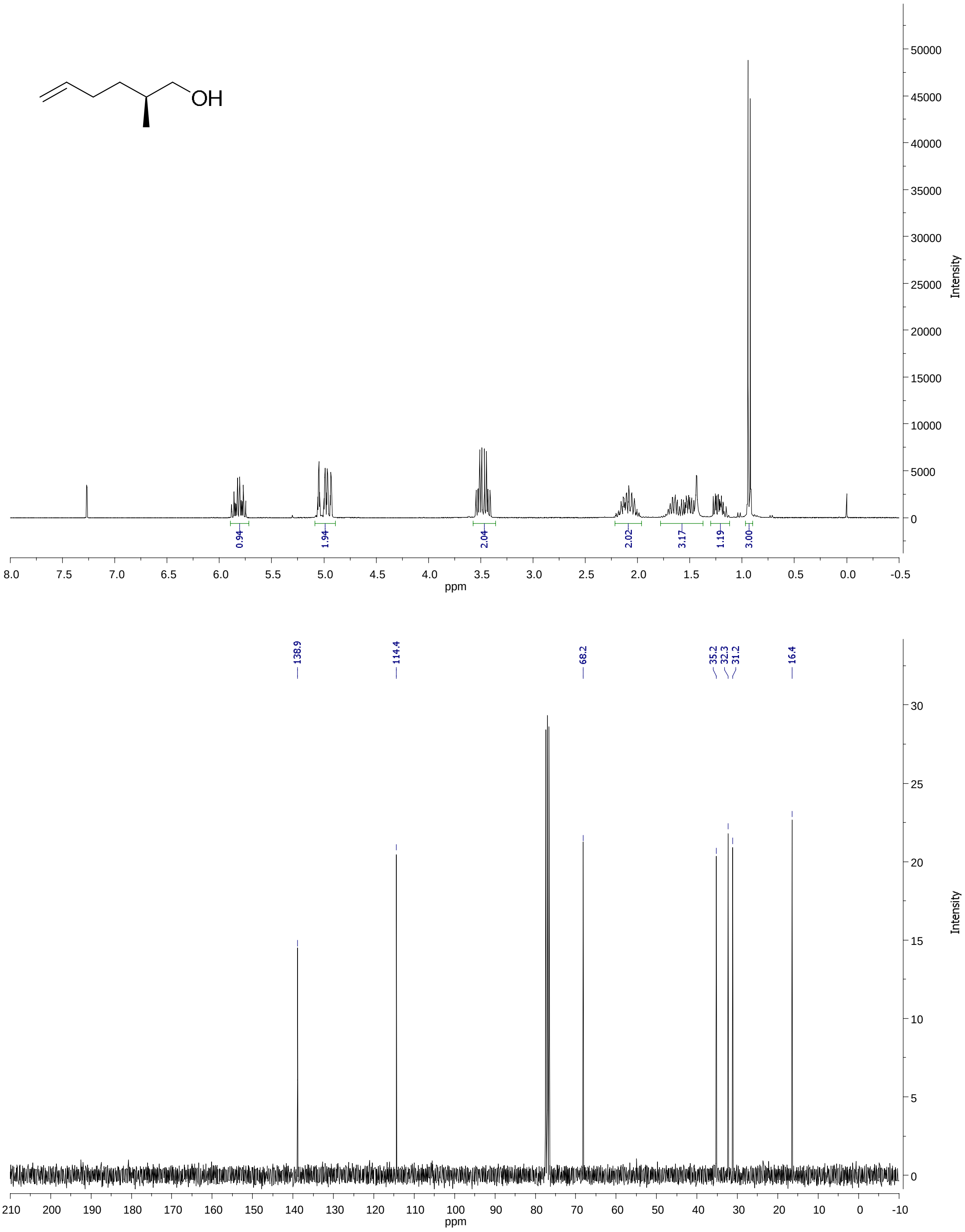

Figure S5: ^{1}H NMR (300 MHz, $CDCl_3$) and ^{13}C NMR (75 MHz, $CDCl_3$) spectrum of (*S*)-2-methylhex-5-en-1-ol (**9**).

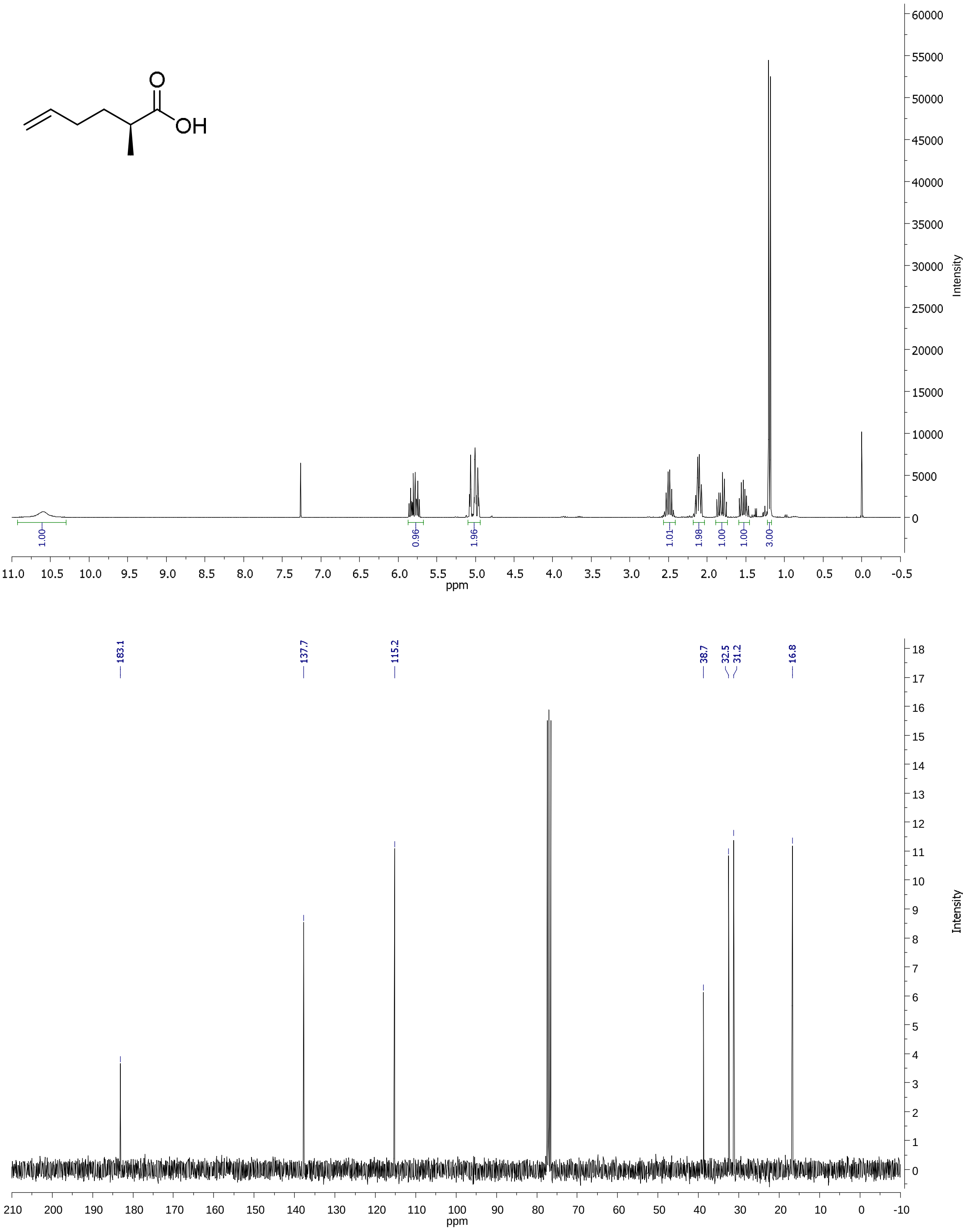

Figure S6: ^{1}H NMR (300 MHz, $CDCl_3$) and ^{13}C NMR (75 MHz, $CDCl_3$) spectrum of (*S*)-2-methylhex-5-enoic acid (**10**).

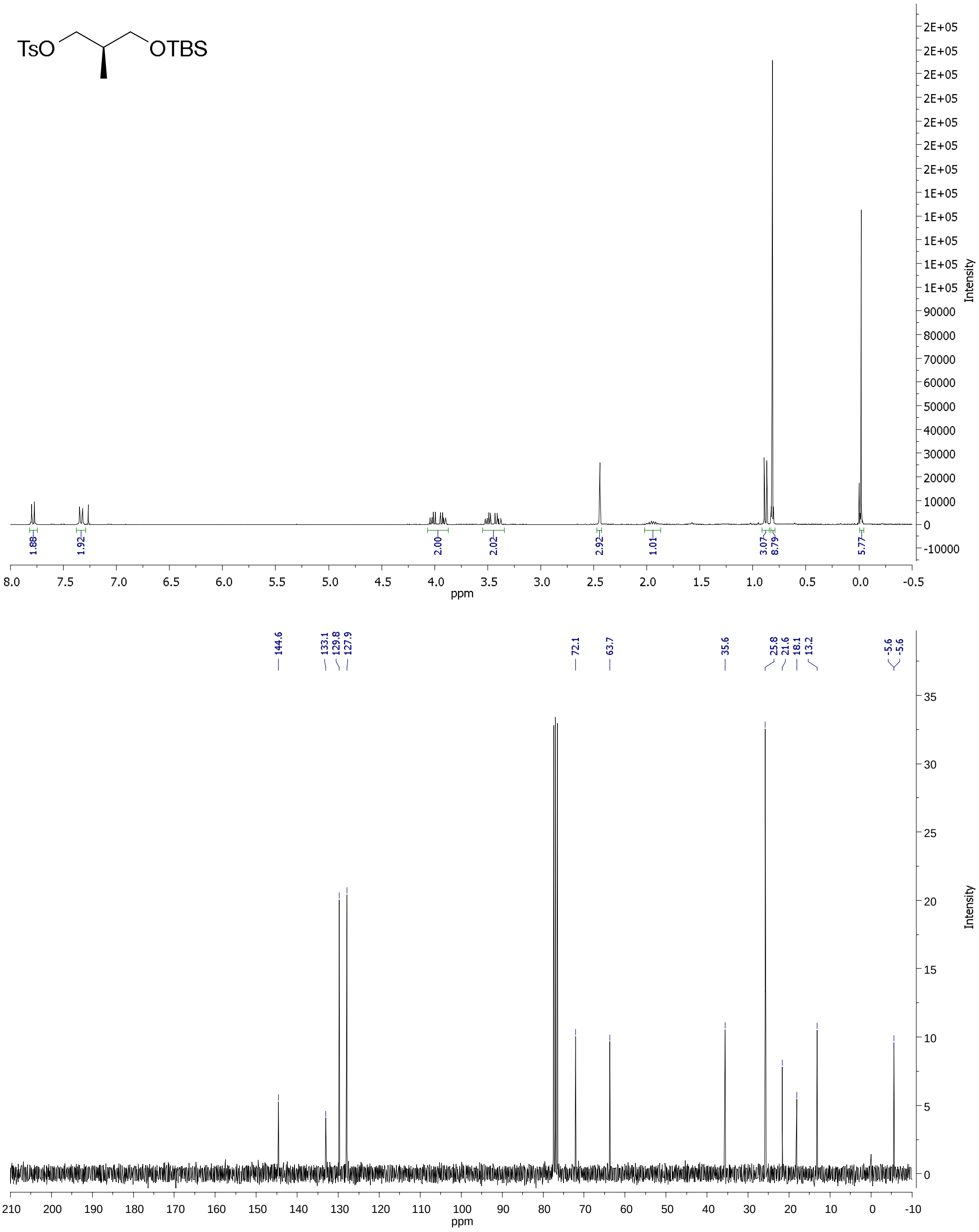

Figure S7: ^{1}H NMR (300 MHz, $CDCl_3$) and ^{13}C NMR (75 MHz, $CDCl_3$) spectrum of (*R*)-3-((*tert*-butyldimethylsilyl)oxy)-2-methylpropyl 4-methylbenzenesulfonate (**11**).

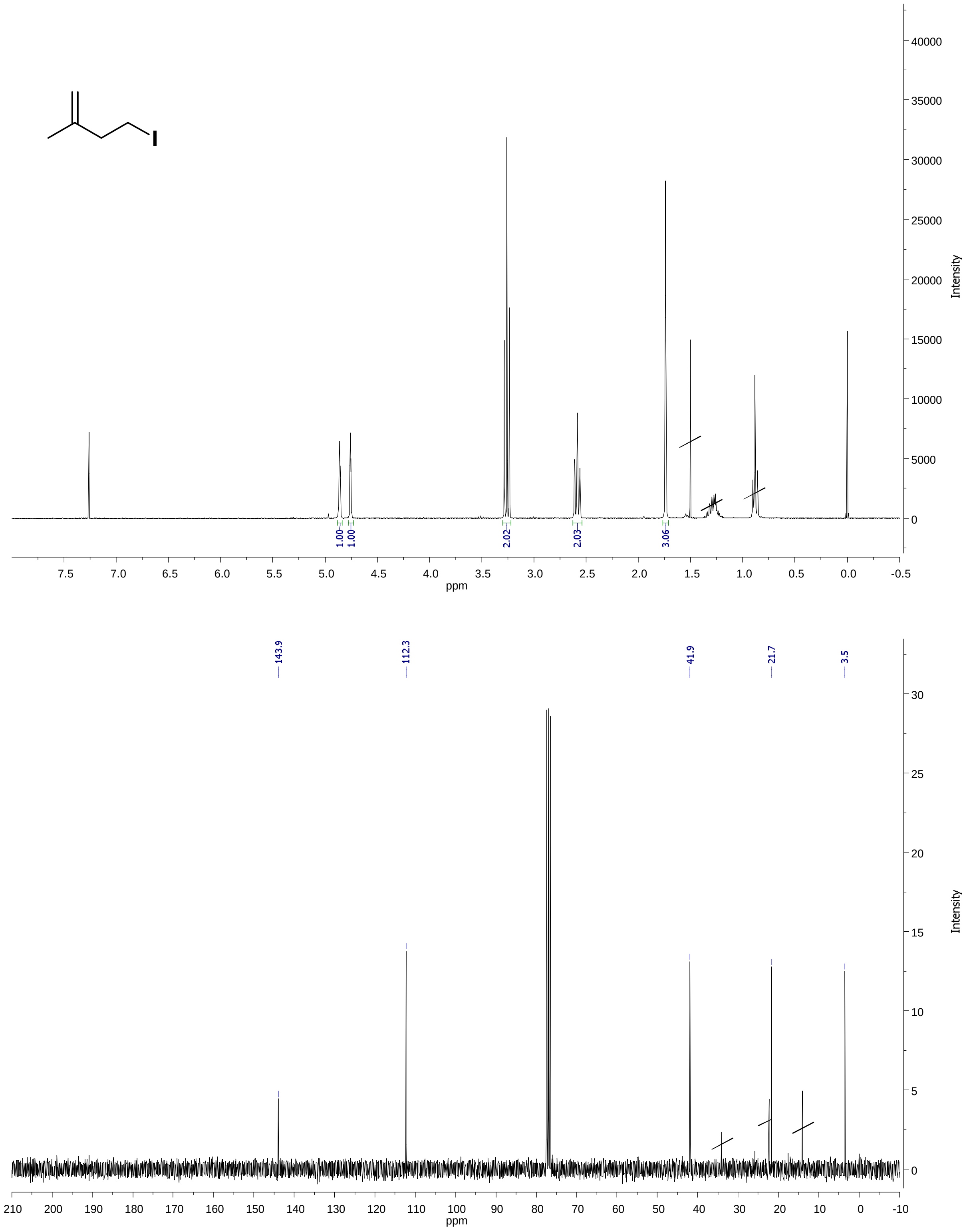

Figure S8: ^{1}H NMR (300 MHz, $CDCl_3$) and ^{13}C NMR (75 MHz, $CDCl_3$) spectrum of 4-iodo-2-methylbut-1-ene (**12**).

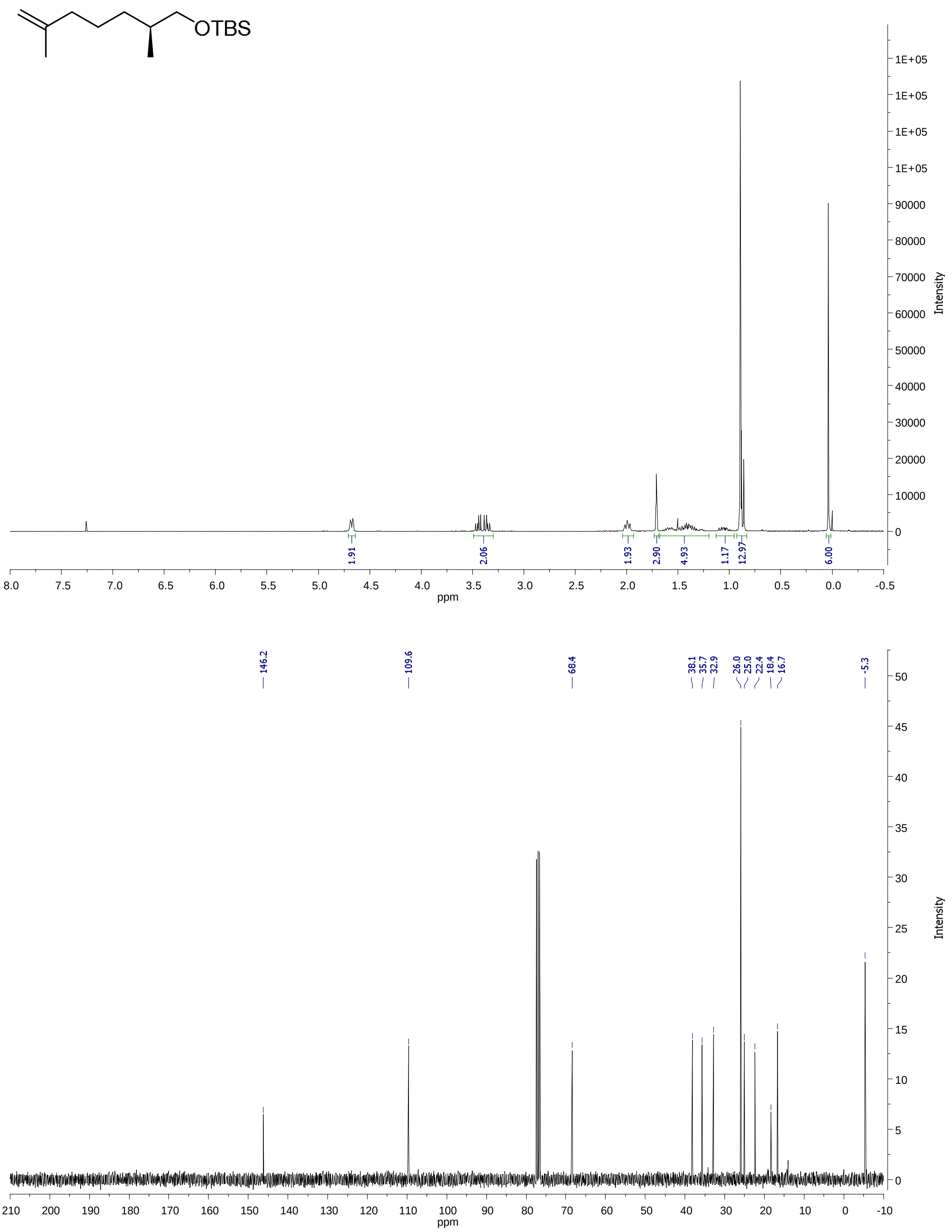

Figure S9: ^{1}H NMR (300 MHz, $CDCl_3$) and ^{13}C NMR (75 MHz, $CDCl_3$) spectrum of (*S*)-*tert*-butyl((2,6-dimethylhept-6-en-1-yl)oxy)dimethylsilane (**13**).

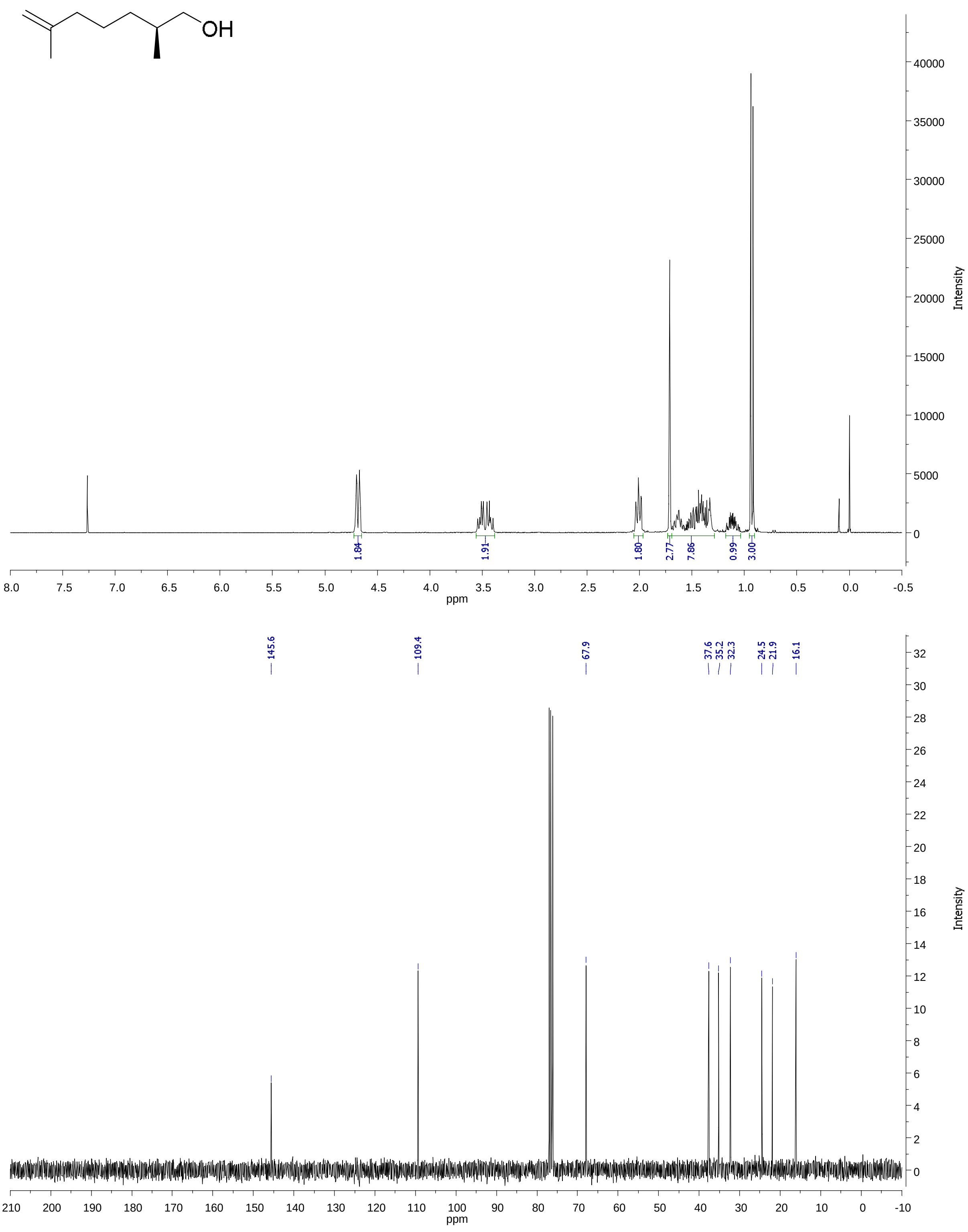

Figure S10: ^{1}H NMR (300 MHz, $CDCl_3$) and ^{13}C NMR (75 MHz, $CDCl_3$) spectrum of (*S*)-2,6-dimethylhept-6-en-1-ol (**14**).

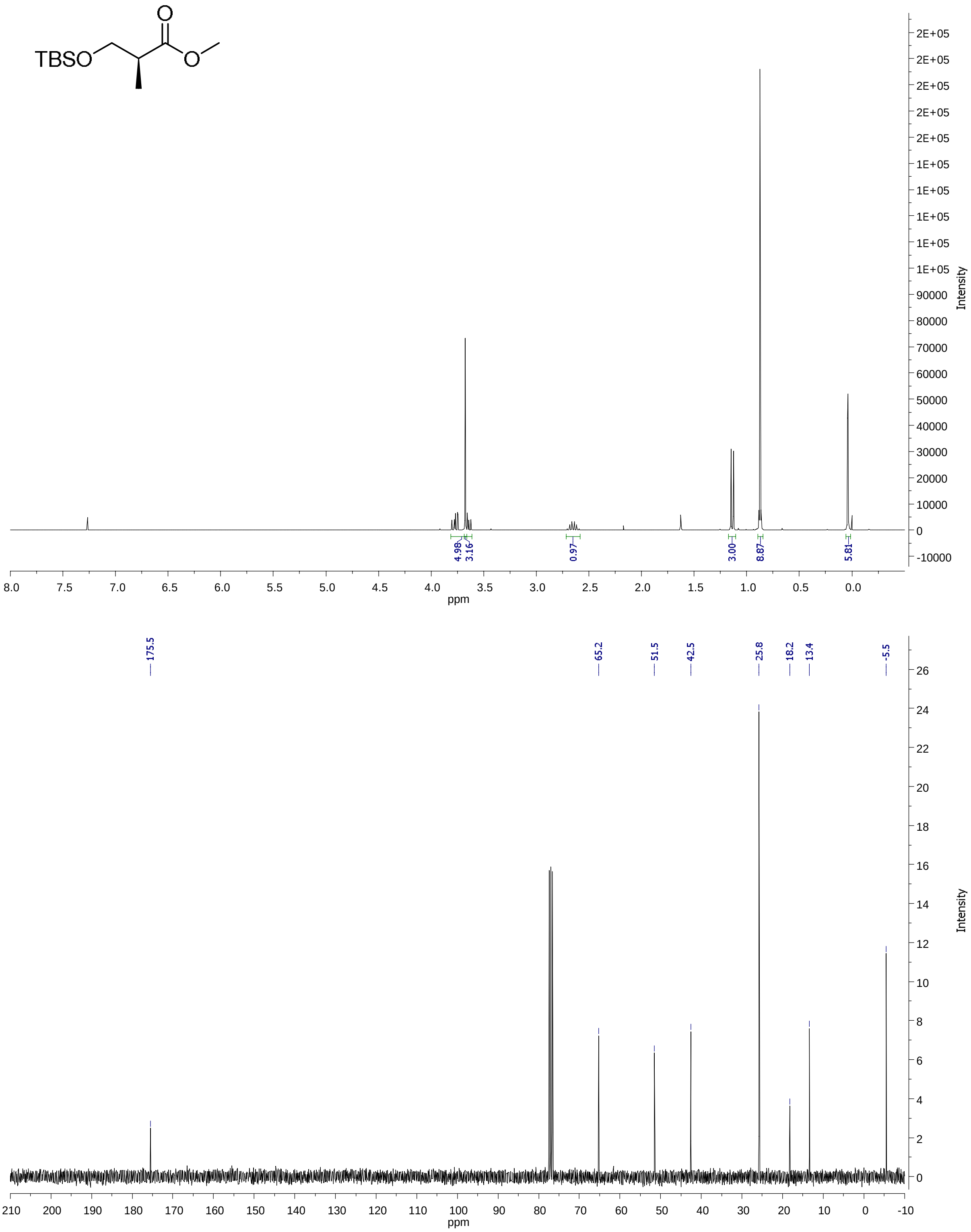

Figure S11: ^{1}H NMR (300 MHz, $CDCl_3$) and ^{13}C NMR (75 MHz, $CDCl_3$) spectrum of (*S*)-3-((*tert*-butyldimethylsilyl)oxy)-2-methylpropanoate (**16**).

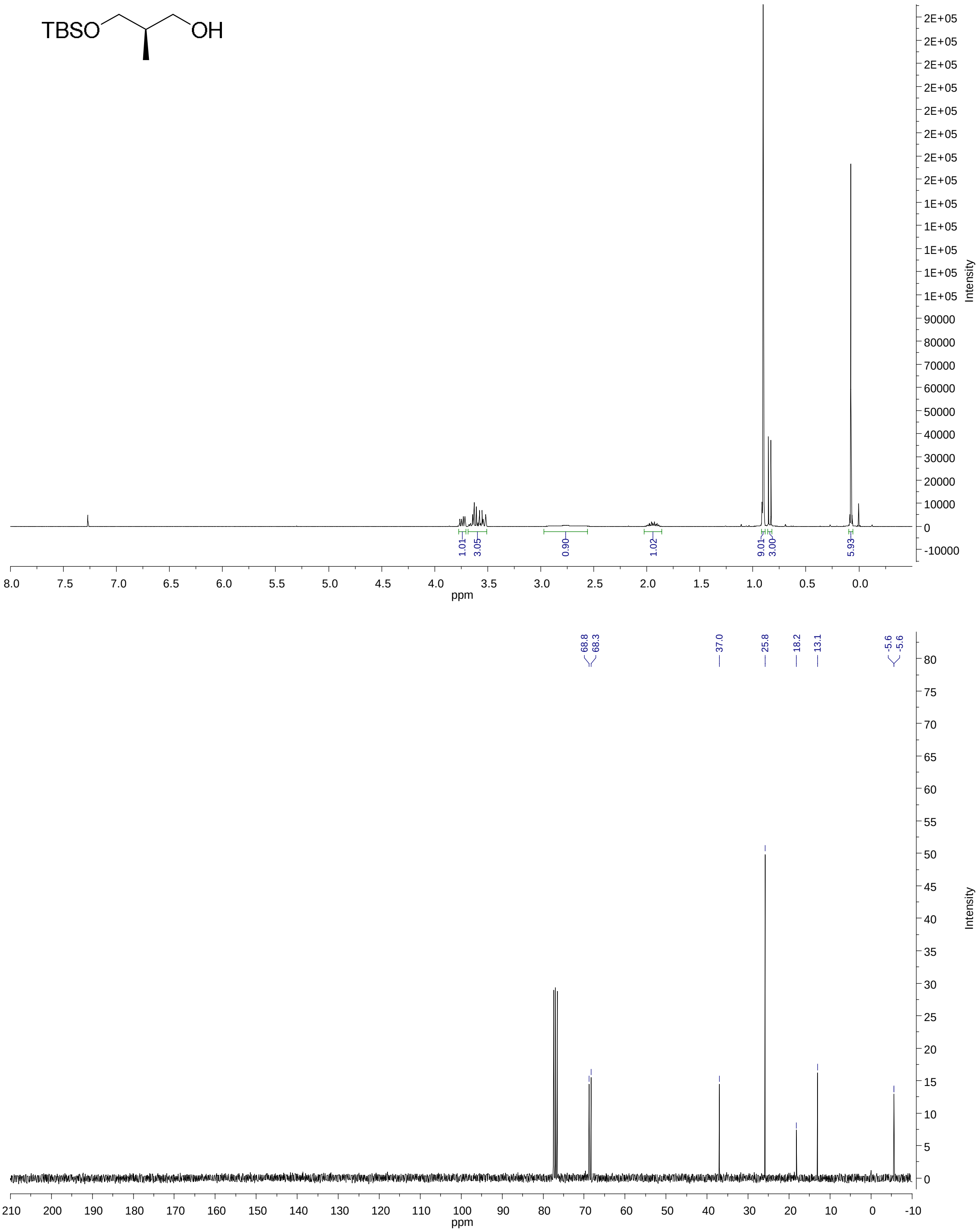

Figure S12: ^{1}H NMR (300 MHz, $CDCl_3$) and ^{13}C NMR (75 MHz, $CDCl_3$) spectrum of (*R*)-3-((*tert*-butyldimethylsilyl)oxy)-2-methylpropan-1-ol (**17**).

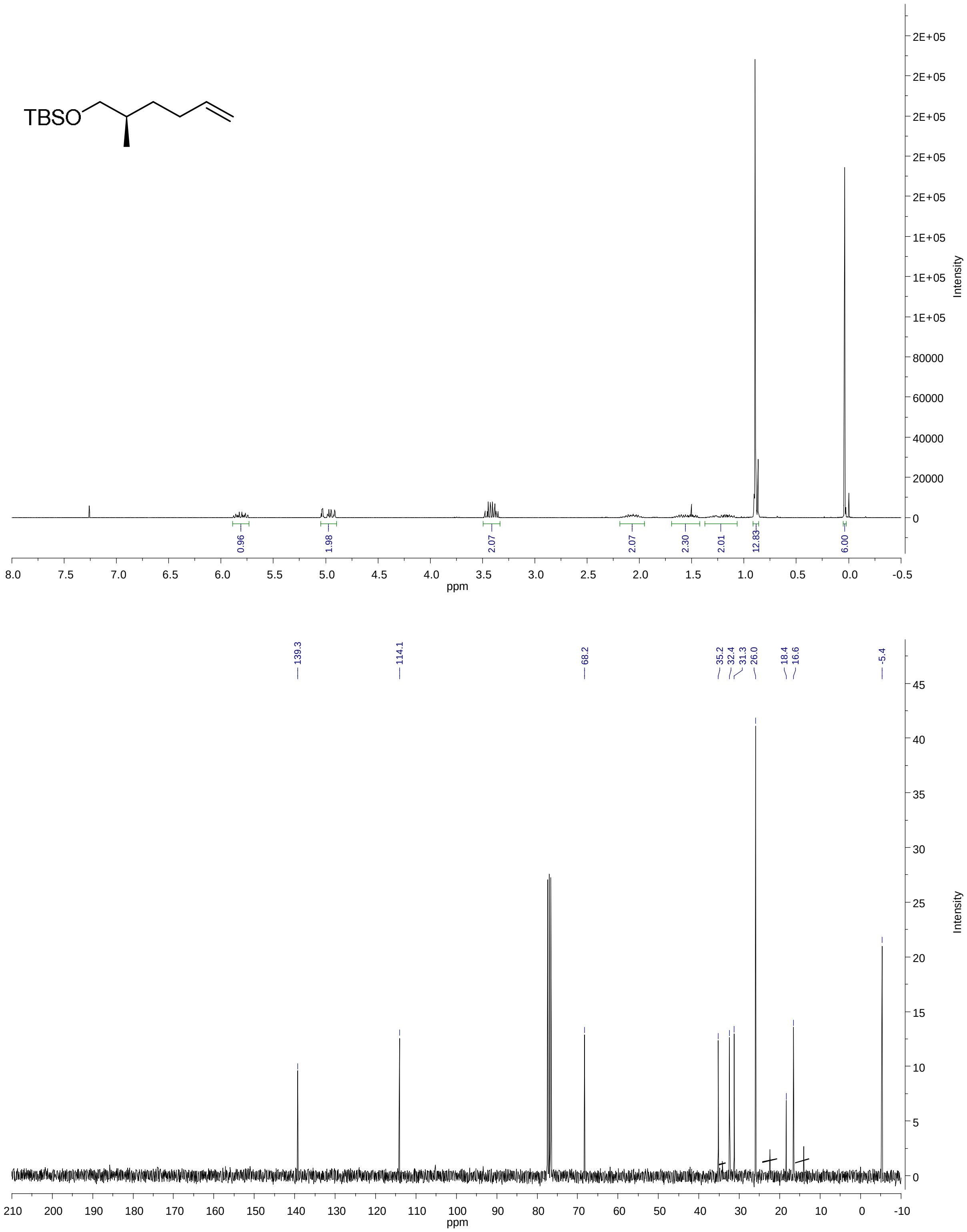

Figure S13: ^{1}H NMR (300 MHz, $CDCl_3$) and ^{13}C NMR (75 MHz, $CDCl_3$) spectrum of (*R*)-*tert*-butyldimethyl((2-methylhex-5-en-1-yl)oxy)silane (**18**).

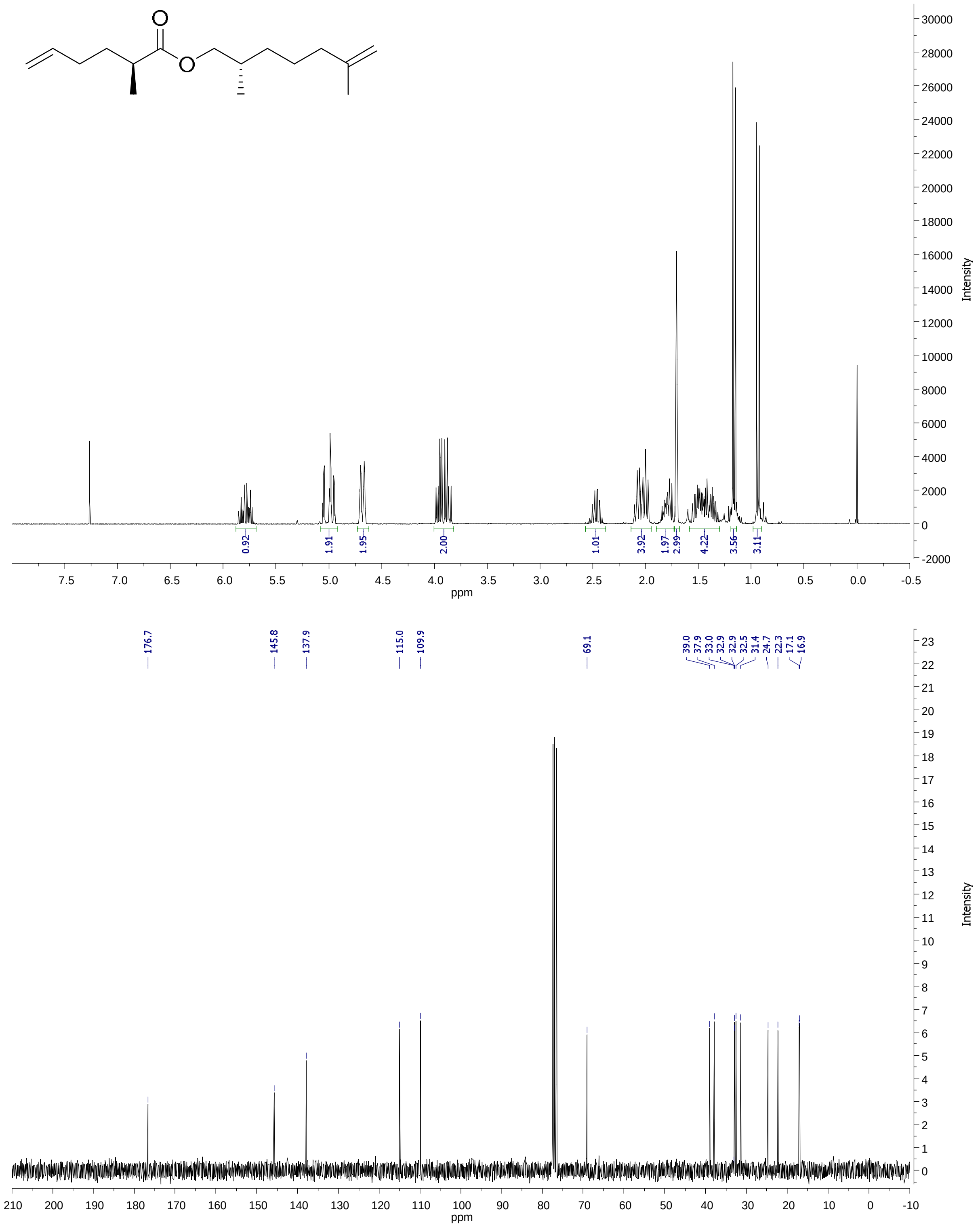

Figure S14: ^{1}H NMR (300 MHz, $CDCl_3$) and ^{13}C NMR (75 MHz, $CDCl_3$) spectrum of (*S*)-2,6-dimethylhept-6-en-1-yl (*S*)-2-methylhex-5-enoate (**15**).

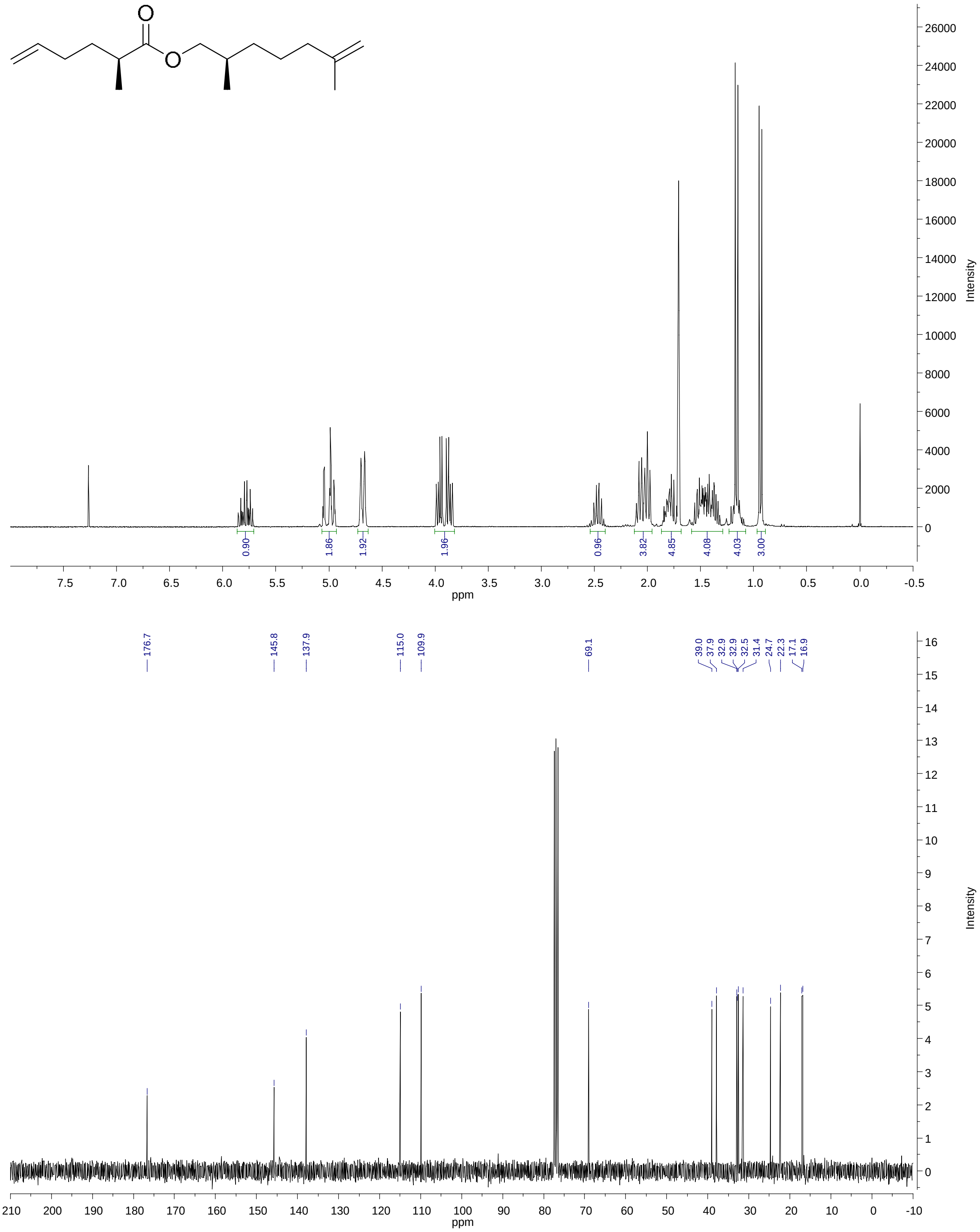

Figure S15: ^{1}H NMR (300 MHz, $CDCl_3$) and ^{13}C NMR (75 MHz, $CDCl_3$) spectrum of (*R*)-2,6-dimethylhept-6-en-1-yl (*S*)-2-methylhex-5-enoate (**15**).

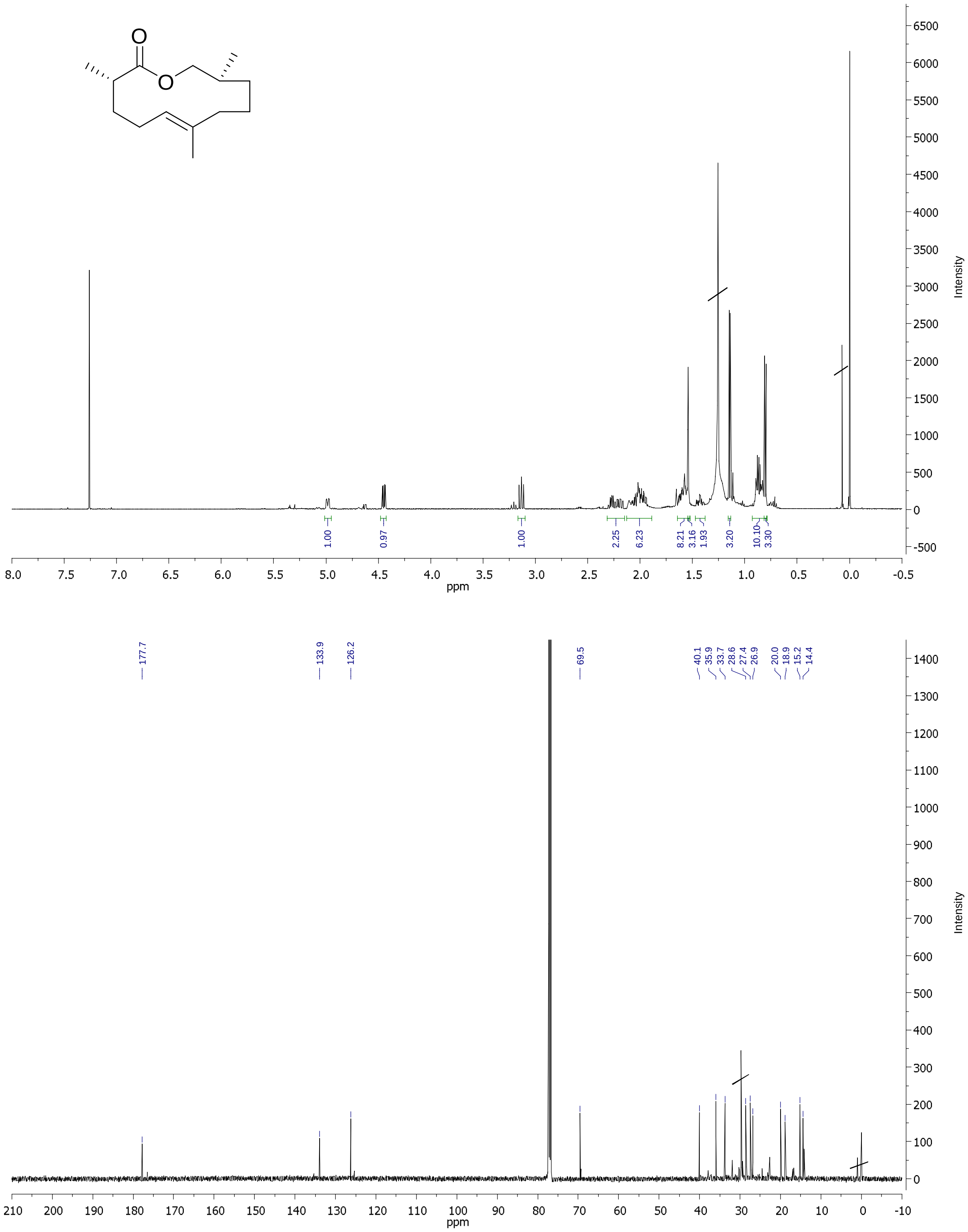

Figure S16: ^{1}H NMR (500 MHz, $CDCl_3$) and ^{13}C NMR (125 MHz, $CDCl_3$) spectrum of (2*S*,5*E*,10*S*)-2,6,10-trimethyl-5-undecen-11-olide (**3**).

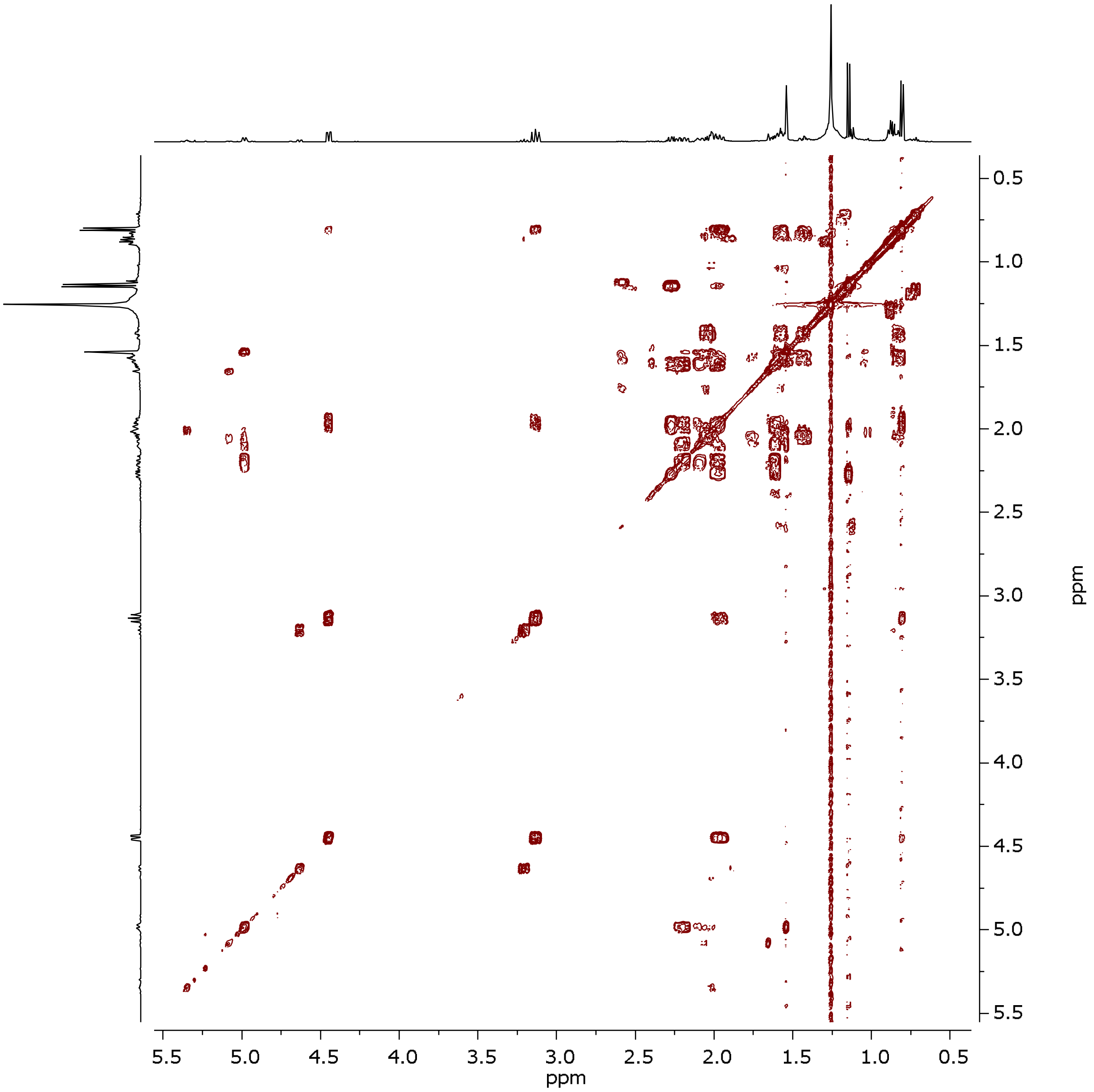

Figure S17: ^{1}H,^{1}H COSY (500 MHz, $CDCl_3$) spectrum of (2*S*,5*E*,10*S*)-2,6,10-trimethyl-5-undecen-11-olide (**3**).

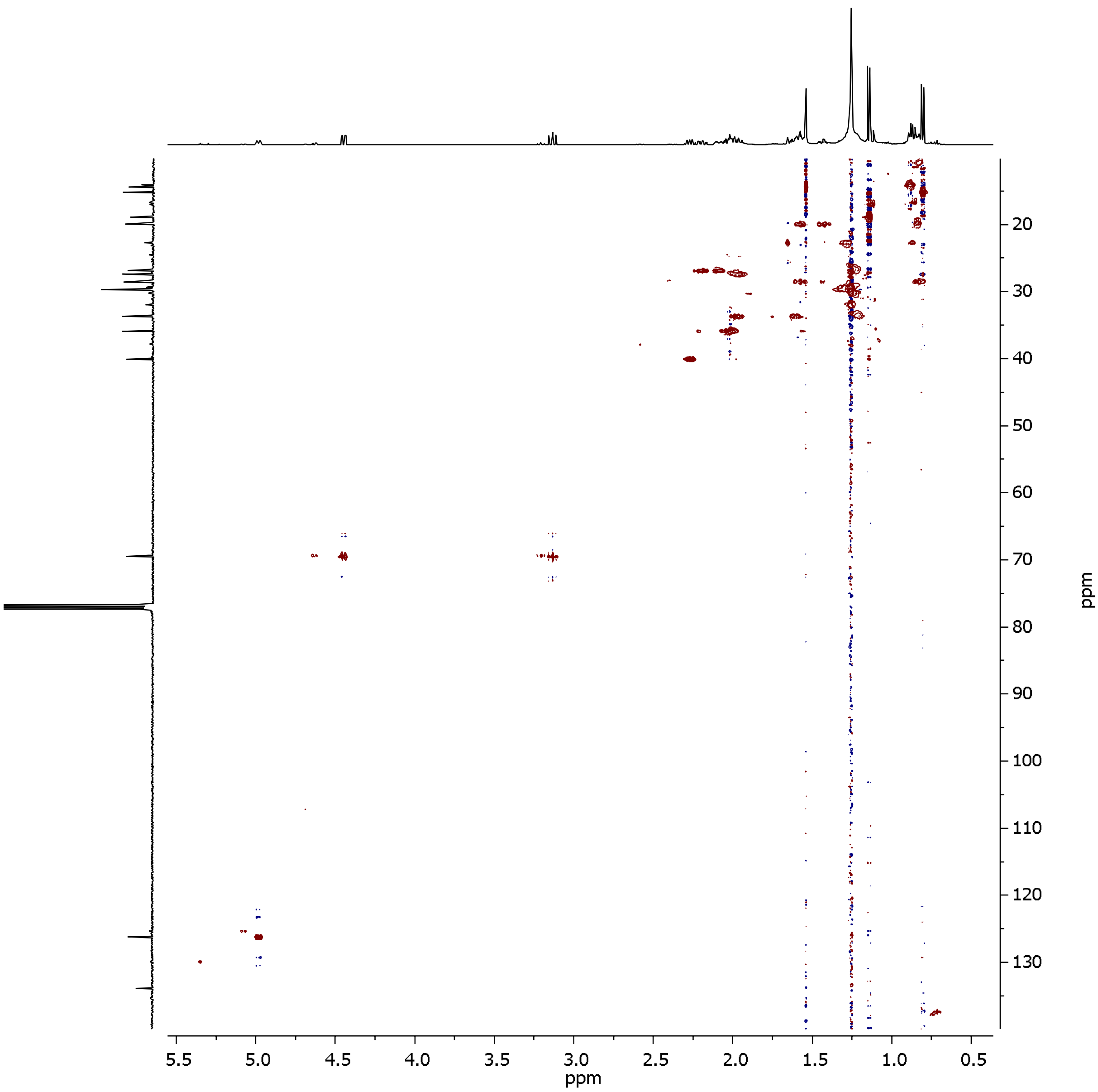

Figure S18: ^{1}H,^{13}C HSQC (500/125 MHz, $CDCl_3$) spectrum of (2*S*,5*E*,10*S*)-2,6,10-trimethyl-5-undecen-11-olide (**3**).

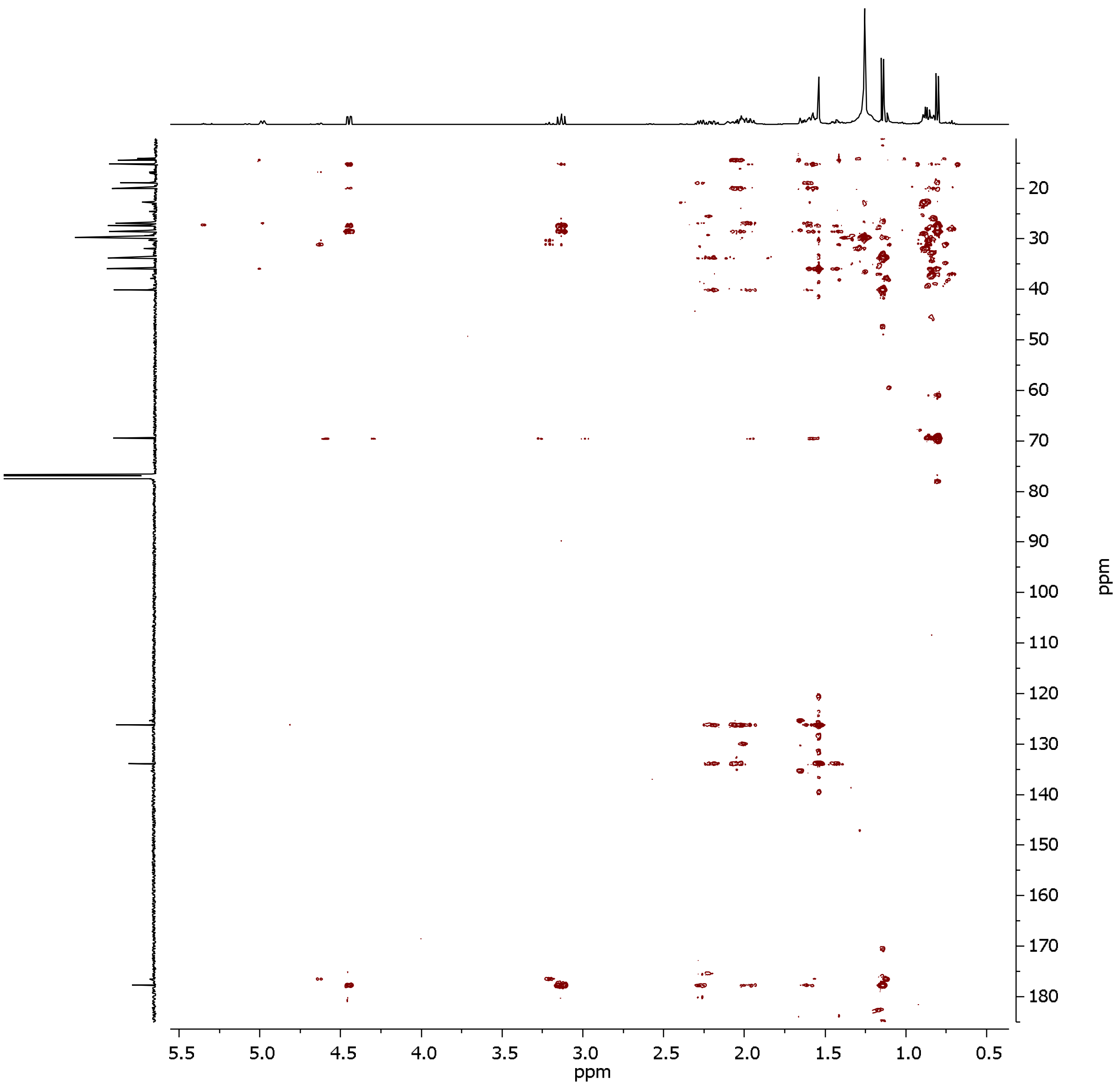

Figure S19: ^{1}H,^{13}C HMBC (500/125 MHz, $CDCl_3$) spectrum of (2*S*,5*E*,10*S*)-2,6,10-trimethyl-5-undecen-11-olide (**3**).

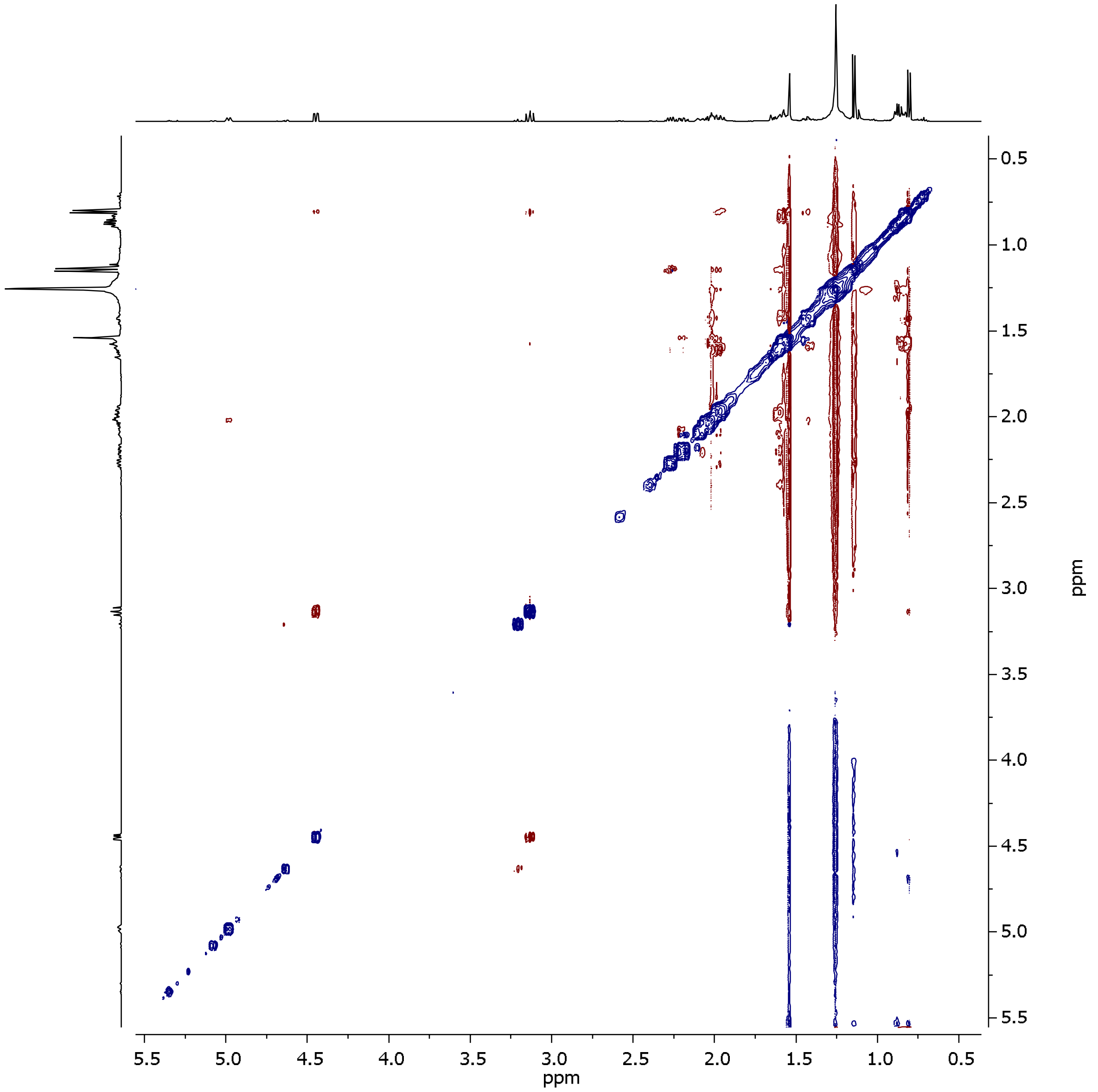

Figure S20: ^{1}H,^{1}H NOESY (500 MHz, $CDCl_3$) spectrum of (2*S*,5*E*,10*S*)-2,6,10-trimethyl-5-undecen-11-olide (**3**).

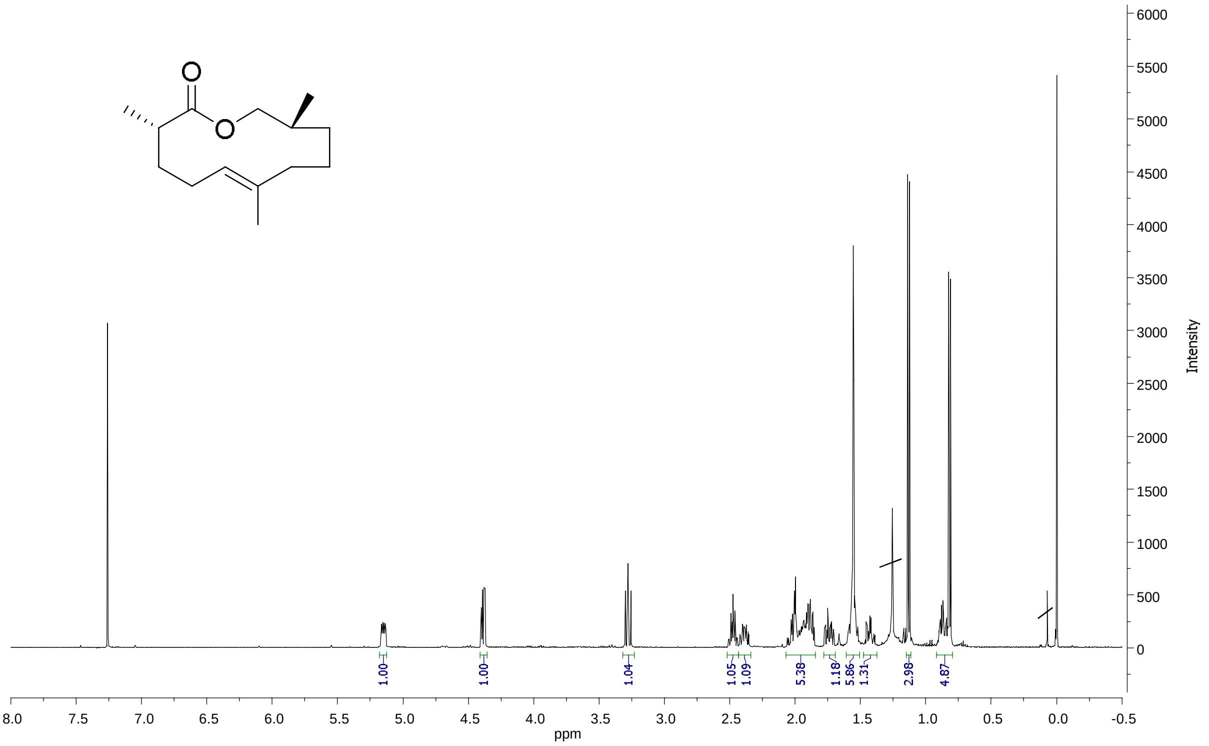

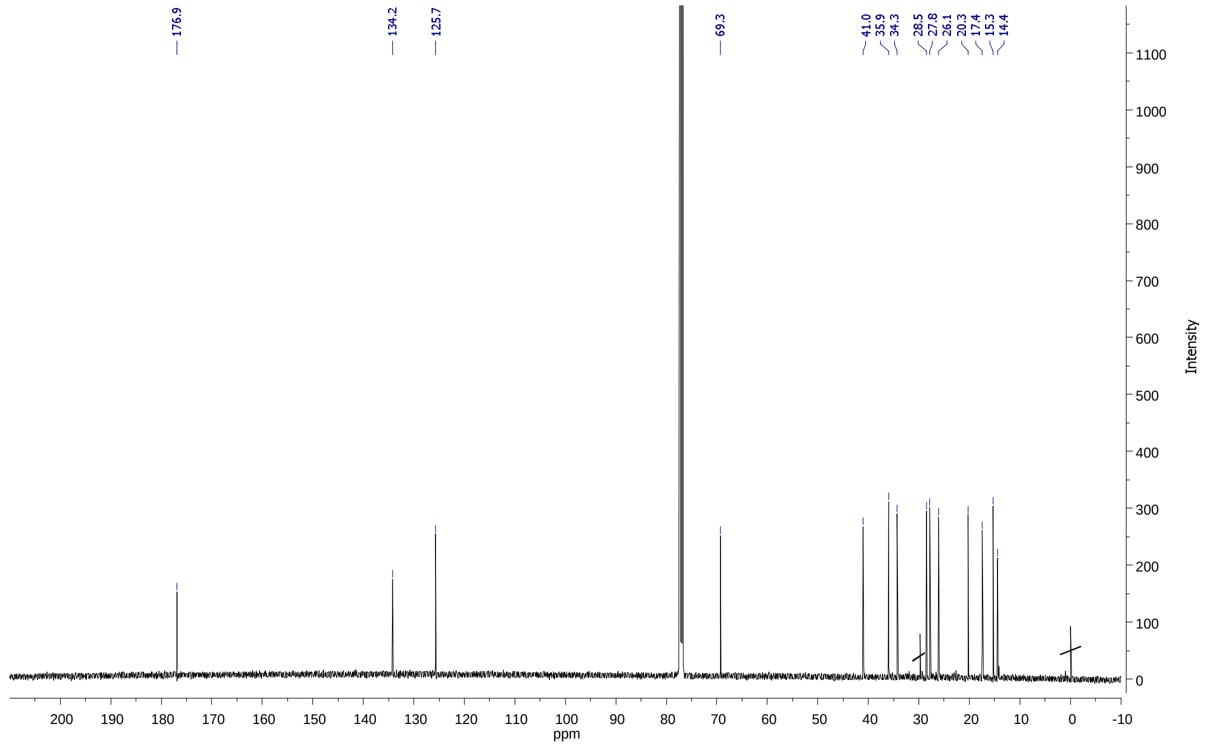

Figure S21: ^{1}H NMR (500 MHz, $CDCl_3$) and ^{13}C NMR (125 MHz, $CDCl_3$) spectrum of (2*S*,5*E*,10*R*)-2,6,10-trimethyl-5-undecen-11-olide (**3**).

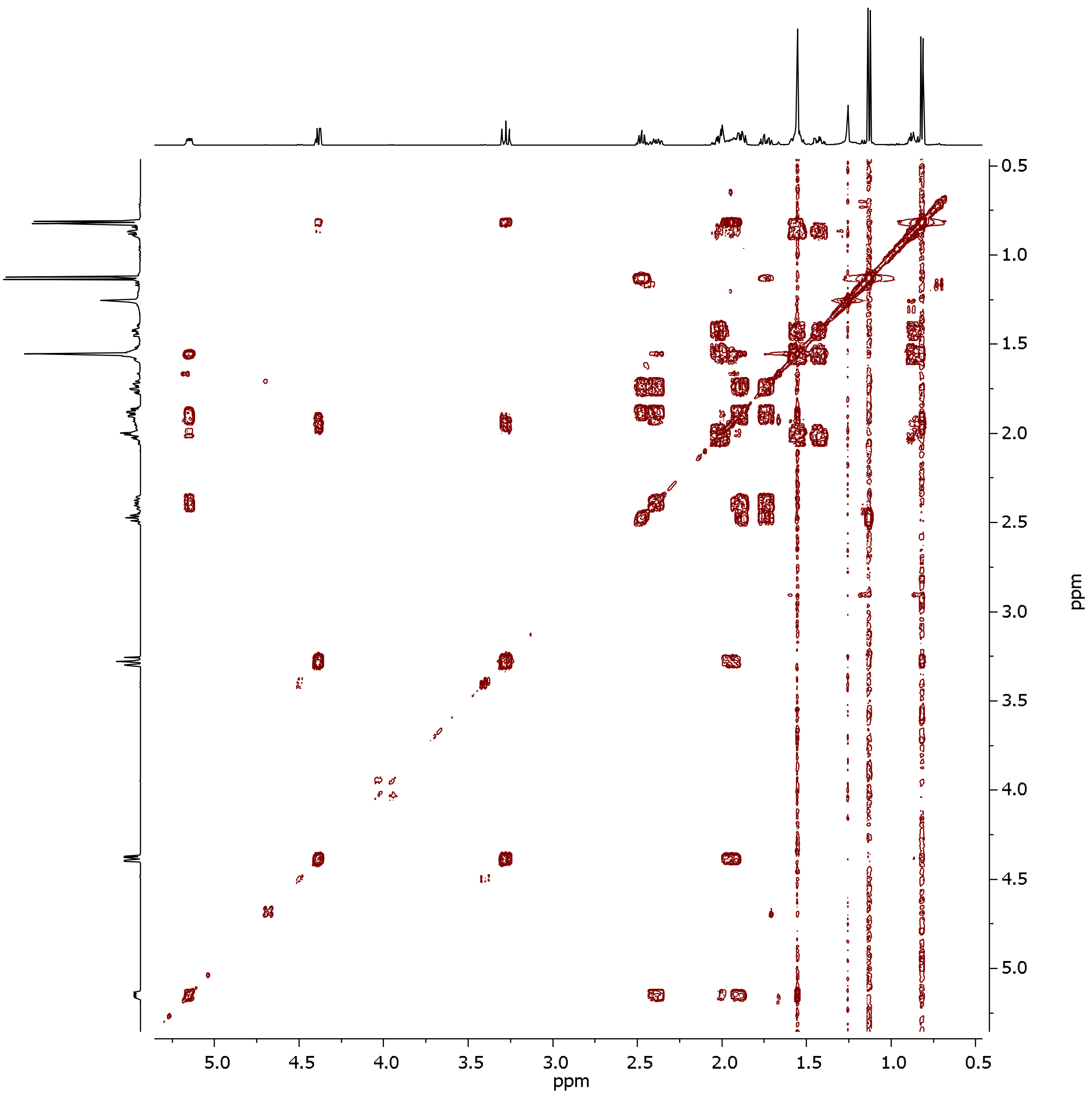

Figure S22: ^{1}H,^{1}H COSY (500 MHz, $CDCl_3$) spectrum of (2*S*,5*E*,10*R*)-2,6,10-trimethyl-5-undecen-11-olide (**3**).

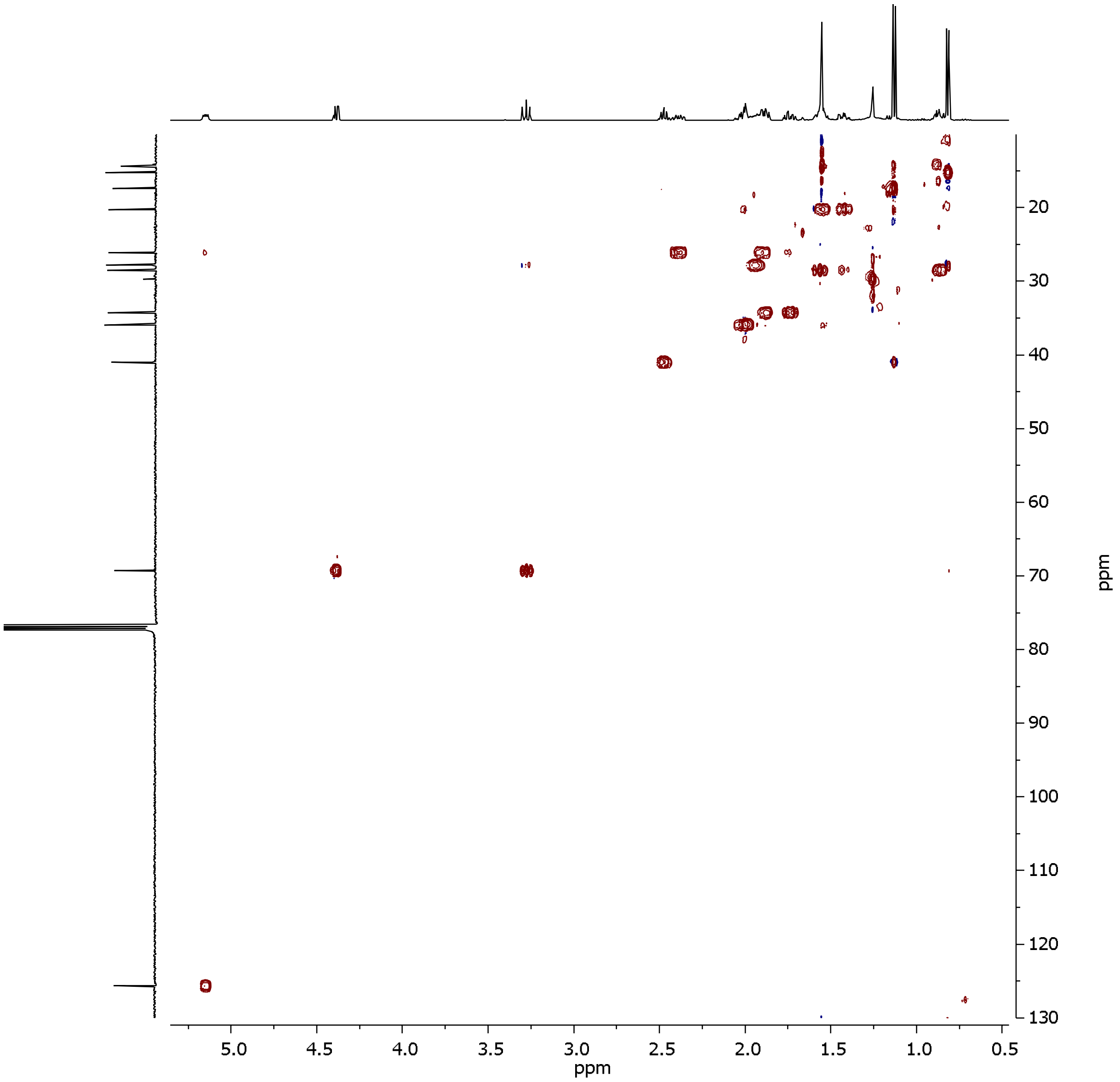

Figure S23: $^{1}H,^{13}C$ HSQC (500/125 MHz, $CDCl_3$) spectrum of (2*S*,5*E*,10*R*)-2,6,10-trimethyl-5-undecen-11-olide (**3**).

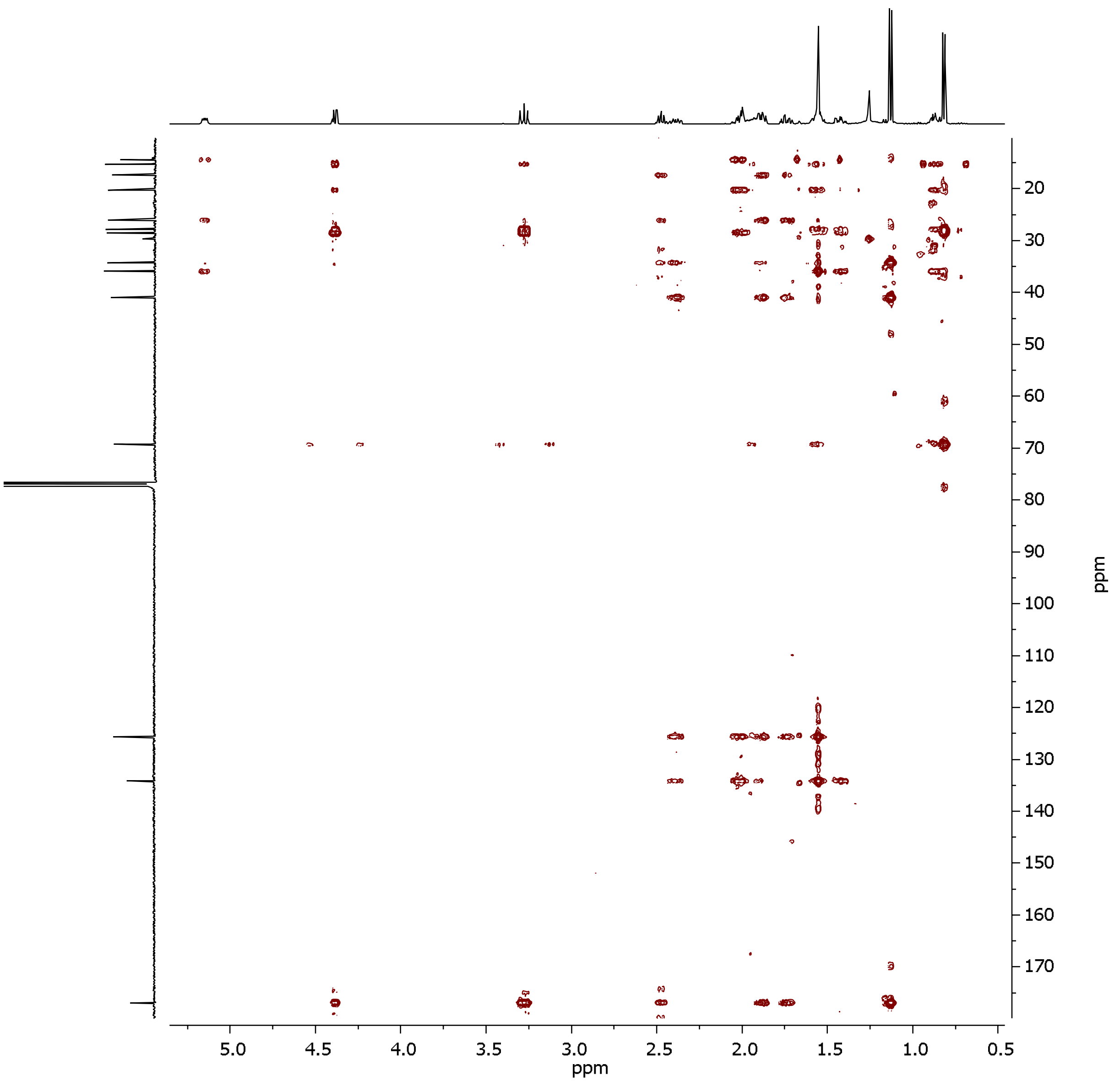

Figure S24: ^{1}H,^{13}C HMBC (500/125 MHz, $CDCl_3$) spectrum of (2*S*,5*E*,10*R*)-2,6,10-trimethyl-5-undecen-11-olide (**3**).

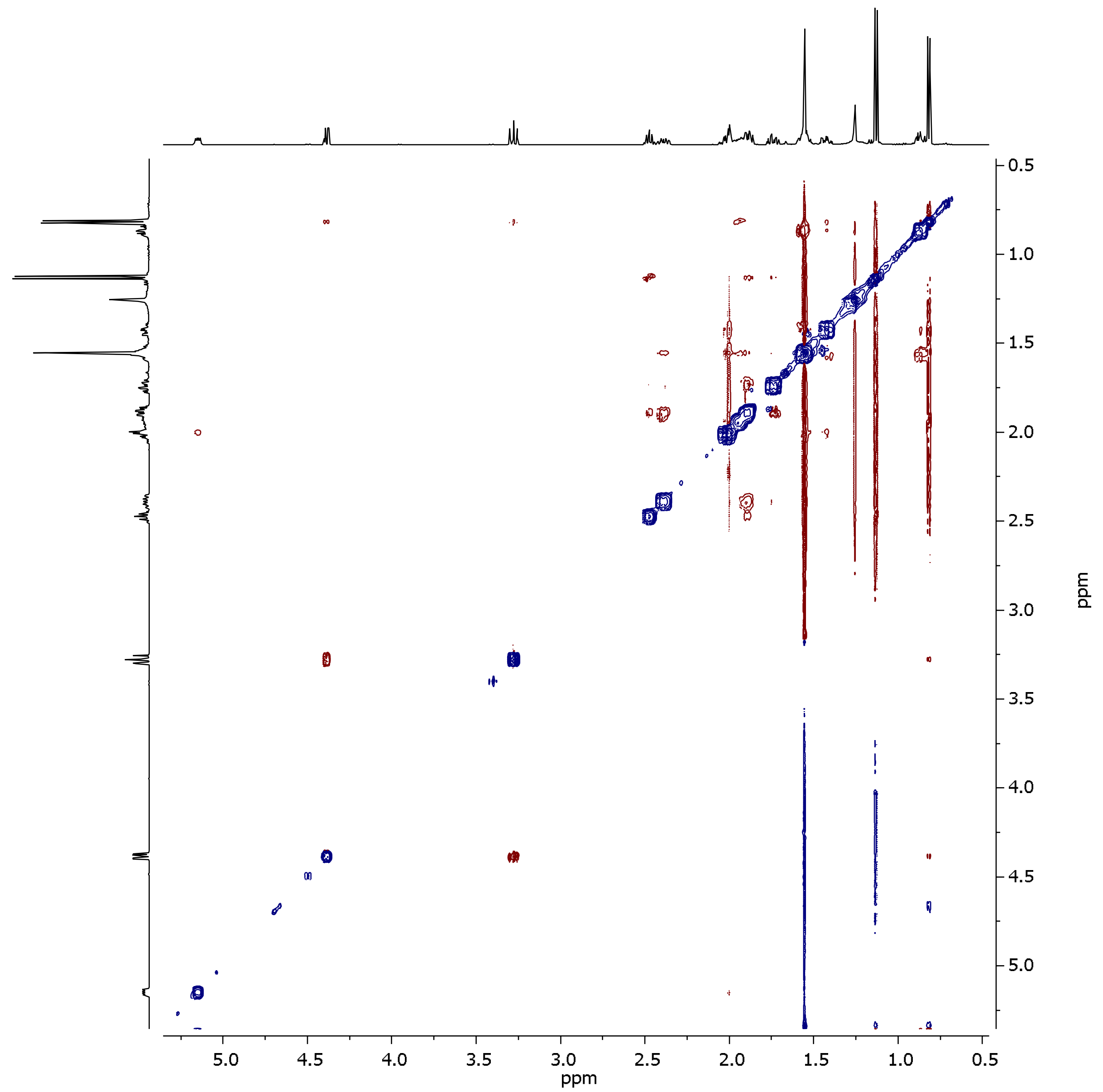

Figure S25: ^{1}H,^{1}H NOESY (500 MHz, $CDCl_3$) spectrum of (2*S*,5*E*,10*R*)-2,6,10-trimethyl-5-undecen-11-olide (**3**).
